# Multi-omics identification of a polyamine metabolism related signature for hepatocellular carcinoma and revealing tumor microenvironment characteristics

**DOI:** 10.3389/fimmu.2025.1570378

**Published:** 2025-04-22

**Authors:** Yuexi Yu, Huiru Liu, Kaipeng Liu, Meiqi Zhao, Yiyan Zhang, Runci Jiang, Fengmei Wang

**Affiliations:** ^1^ Department of gastroenterology &hepatology, Tianjin First Center Hospital, Tianjin Key Laboratory for Organ Transplantation, Tianjin Key Laboratory of Molecular Diagnosis and Treatment of Liver Cancer, Tianjin Medical University, Tianjin, China; ^2^ Department of Hepatobiliary Oncology, Liver Cancer Center, Tianjin Medical University Cancer Institute & Hospital, National Clinical Research Center for Cancer, Key Laboratory of Cancer Prevention and Therapy, Tianjin’s Clinical Research Center for Cancer, Tianjin Medical University, Tianjin, China; ^3^ Department of gastroenterology &hepatology, Tianjin First Center Hospital, Tianjin Key Laboratory for Organ Transplantation, Tianjin Key Laboratory of Molecular Diagnosis and Treatment of Liver Cancer, Nankai University, Tianjin, China

**Keywords:** hepatocellular carcinoma, multi-omics analysis, single-cell RNA sequencing, polyamine metabolism, immune therapy, machine learning

## Abstract

**Background:**

Accumulating evidence indicates that elevated polyamine levels are closely linked to tumor initiation and progression. However, the precise role of polyamine metabolism in hepatocellular carcinoma (HCC) remains poorly understood.

**Methods:**

We conducted differential expression analysis on bulk RNA sequencing data from The Cancer Genome Atlas (TCGA) and Gene Expression Omnibus (GEO) to identify 65 polyamine metabolism-related genes. By employing unsupervised consensus clustering, AddModuleScore, single-sample gene set enrichment analysis (ssGSEA), and weighted gene co-expression network analysis (WGCNA), we identified polyamine metabolism-related genes at both the bulk RNA-seq and single-cell RNA-seq (scRNA-seq) levels. Utilizing 101 machine learning algorithms, we constructed a polyamine metabolism-related signature (PMRS) and validated its predictive power across training, testing, and external validation cohorts. Additionally, we developed a prognostic nomogram model by integrating PMRS with clinical variables. To explore immune treatment sensitivity, we assessed tumor mutation burden (TMB), tumor immune dysfunction and exclusion (TIDE) score, mutation frequency, and immune checkpoint genes expression. Immune cell infiltration was analyzed using the CIBERSORT algorithm. Finally, RT-qPCR experiments were conducted to validate the expression of key genes.

**Results:**

Using 101 machine learning algorithms, we established a polyamine metabolism-related signature comprising 9 genes, which exhibited strong prognostic value for HCC patients. Further analysis revealed significant differences in clinical features, biological functions, mutation profiles, and immune cell infiltration between high-risk and low-risk groups. Notably, TIDE analysis and immune phenotype scoring (IPS) demonstrated distinct immune treatment sensitivities between the two risk groups. RT-qPCR validation confirmed that these 9 genes were highly expressed in normal cells but significantly downregulated in tumor cells.

**Conclusions:**

Our study developed a polyamine metabolism-based prognostic risk signature for HCC, which may provide valuable insights for personalized treatment strategies in HCC patients.

## 1 Introduction

Hepatocellular carcinoma (HCC) is the most prevalent primary liver cancer and the second leading cause of cancer-related deaths worldwide (1). In recent years, great progresses have been made in HCC therapy, but the prognosis of HCC patients remains poor, because of high rate of recurrence and being diagnosed at an advanced stage (2). Conventional treatment options for HCC, including surgery, chemotherapy, and radiofrequency ablation, have been complemented by significant advances in targeted immunotherapy combinations (3). The IMbrave150 clinical trial results showed that, compared to traditional first-line chemotherapy with sorafenib, the combination of atezolizumab (an anti-PD-L1 antibody) and bevacizumab (an anti-VEGF antibody) significantly improved advanced-stage HCC patient outcomes (4). Despite these advancements, the efficacy of these therapies remains limited for patients with advanced-stage HCC, who often experience high recurrence rates and ultimately face a worsened prognosis (4, 5).

Polyamines, including putrescine, spermidine (SPM), and spermine (SPM), are molecules derived from ornithine through a decarboxylation process catalyzed by ornithine decarboxylase (ODC). Putrescine is subsequently converted into spermidine and spermine through the catalytic action of spermidine synthase (SRM) and spermine synthase (SMS), respectively (6). These polyamines play essential roles in key cellular processes such as proliferation, differentiation, and apoptosis, and are closely associated with tumorigenesis (7). Moreover, S-adenosylmethionine decarboxylase (AMD1) is a critical enzyme involved in spermidine synthesis. AMD1 promotes the stemness of HCC cells through mRNA demethylation mediated by fat mass and obesity-associated protein (FTO). When AMD1 is upregulated in HCC tissues, it is often linked to poor prognosis (8). Additionally, studies have shown that oncogenes such as MYC and BRAF can significantly regulate polyamine levels in cancer cells (9).

The tumor microenvironment (TME) is a complex, localized milieu consisting of tumor cells, non-tumor cells, the extracellular matrix (ECM), blood vessels, immune cells, signaling molecules, and other components. The establishment of an immune-suppressive microenvironment is closely associated with the immune evasion of tumor cells (10). Within the TME, polyamine levels in immune cells are typically elevated (6). Research by Chia et al. demonstrated that inhibiting polyamine metabolism in cancer could suppress tumor growth, possibly by promoting increased T-cell infiltration and macrophage-driven pro-inflammatory phenotypes (11). Studies have suggested that targeting polyamine metabolism can modify the TME, thereby enhancing the immune system’s ability to combat tumors. Inhibition of polyamine synthesis has been shown to significantly slow tumor growth and improve the efficacy of immunotherapy (12).

Currently, the precise molecular mechanisms through which polyamine metabolism-related biomarkers influence HCC remain unclear. In this study, we employed multi-omics analysis to investigate the role of polyamine metabolism features in HCC. By integrating bulk RNA sequencing and single-cell RNA sequencing data, we identified key genes associated with polyamine metabolism. We then applied machine learning techniques to develop a risk score model for HCC patients, further elucidating the tumor immune microenvironment characteristics across different risk profiles.

## 2 Materials and methods

### 2.1 Data sources

We downloaded transcriptomic data, mutation data, copy number variation (CNV) data, and clinical information for 424 HCC samples from The Cancer Genome Atlas (TCGA, https://portal.gdc.cancer.gov/projects/TCGA), including 50 normal samples and 374 tumor samples. Additionally, we obtained the GSE14520 dataset from the Gene Expression Omnibus (GEO, https://www.ncbi.nlm.nih.gov/geo/), which contains 220 normal samples and 225 tumor samples. The ICGC-LIRI dataset from the International Cancer Genome Consortium (ICGC, https://dcc.icgc.org/) was also retrieved, including 177 normal samples and 212 tumor samples (**Supplementary Tables S1–S3**). Single-cell RNA sequencing data were obtained from the GSE242889 dataset, which consists of 5 normal samples and 5 tumor samples.

### 2.2 Differentially expressed genes (DEGs) associated with polyamine metabolism in HCC

Genes related to polyamine metabolism (PMRG) were sourced from GeneCards (https://www.genecards.org/) and the MSigDB database (http://www.gseamsigdb.org/), totaling 814 genes (**Supplementary Table S4**). We performed differential expression analysis between tumor and normal tissues in the TCGA-LIHC and GSE14520 datasets using the R package “limma” (**Supplementary Table S5**). After filtering, 65 differentially expressed genes associated with polyamine metabolism in HCC were identified, with the screening criteria set at an adjusted p-value < 0.05 and |log2 fold change| > 1 (13).

### 2.3 Consensus clustering analysis

We performed consensus clustering of the differentially expressed genes associated with polyamine metabolism in HCC using the R package “ConsensusClusterPlus” and the K-means algorithm. The cases were grouped into multiple clusters based on specific markers or features. To determine the optimal number of clusters, we conducted 1,000 resamplings using 80% of the samples. Kaplan-Meier survival curve analysis was then performed to compare overall survival (OS) rates of HCC patients across different clusters. Finally, univariate Cox regression analysis identified 33 genes significantly associated with HCC prognosis, which were selected for further analysis (p-value < 0.05).

### 2.4 Single-cell RNA sequencing data collection and processing

We preprocessed the GSE242889 dataset using the “Seurat” package. Initially, cells with fewer than 250 or more than 7,000 expressed genes, or those with more than 15% unique molecular identifiers (UMIs) derived from mitochondrial genes, were excluded as low-quality cells. Next, we removed mitochondrial, ribosomal, and hemoglobin genes from the dataset. A total of 37,330 cells were retained for further analysis. To correct for batch effects between datasets, we applied canonical correlation analysis (CCA) (14). Using the “FindVariableFeatures” function with default parameters from the Seurat package, we identified the top 2,000 most variable genes. Cells were then clustered using the “FindClusters” and “FindNeighbors” functions, and the resulting clusters were visualized using the Uniform Manifold Approximation and Projection (UMAP) method. Cluster annotation was performed using a combination of the SingleR package and manual annotation (15). The annotation results for each cell cluster are shown in **Supplementary Table S6**. We quantified the PMG score for each cell using the “AddModuleScore” function in Seurat. To identify differentially expressed genes (DEGs) between high and low PMG score groups, we applied the “FindMarkers” function from the Seurat package and used the Wilcoxon test (p.adj < 0.05) for DEG selection. Genes differentially expressed between cells with high and low PMG scores were associated with polyamine metabolism.

### 2.5 Single-sample gene set enrichment analysis and gene set enrichment analysis

Single-Sample Gene Set Enrichment Analysis (ssGSEA) is a method based on gene expression data to assess the enrichment of specific gene sets within individual samples. Using the ssGSEA method, we calculated the PMG scores(PMGS) for the TCGA-LIHC samples (16).

To identify potential biological pathways associated with these features, we first calculated GSVA scores for 50 hallmark pathways and analyzed significant differences in pathway activity between the high-risk and low-risk groups using the “limma” package. Additionally, to further investigate the biological processes (BP), cellular components (CC), and molecular functions (MF) involved in different risk subgroups, we conducted GSEA using the “DOSE,” “clusterProfiler,” and “GseaVis” R packages on the GO gene sets (c5.go.v7.5.1.symbols.gmt). The screening criteria were set to a False Discovery Rate (FDR) < 0.05 and |Normalized Enrichment Score (NES)| > 1 (17).

### 2.6 Weighted gene co-expression network analysis

Weighted Gene Co-Expression Network Analysis (WGCNA) is an unsupervised learning method based on gene co-expression patterns. It constructs a weighted gene co-expression network to identify cooperative expression relationships between genes and organizes them into distinct modules. These modules can then be correlated with clinical phenotypes, disease states, or other characteristics.To identify gene sets associated with polyamine metabolism, we performed WGCNA using the “WGCNA” R package (18). Based on the TCGA-LIHC bulk transcriptome data, we first calculated an appropriate soft-thresholding power (β) to ensure that the network adhered to the scale-free topology property. The weighted adjacency matrix was then transformed into a Topological Overlap Matrix (TOM), and dissTOM (dissimilarity) was computed. we used the dynamic tree cut algorithm to cluster the genes and identify modules. Finally, we selected the modules most strongly correlated with the PMG scores for further analysis.

### 2.7 Development and validation of HCC prognostic features

We used the “limma” R package to perform differential analysis between the high PMG score group and low PMG score group in the TCGA bulk RNA-seq data. The screening criteria were set at a False Discovery Rate (FDR) < 0.05 and log2 fold change (FC) > 1. we conducted an intersection analysis between the differentially expressed genes (DEGs) and genes from the PMG score-related modules identified by WGCNA, with the intersected genes used for subsequent analysis.

To construct a robust prognostic model with high predictive accuracy, we followed these steps based on previous studies (19, 20).

#### 2.7.1 Univariate Cox regression analysis

We first performed univariate Cox regression analysis to identify genes with prognostic significance in the TCGA-LIHC dataset.

#### 2.7.2 Dataset splitting and model construction

TCGA-LIHC was used as the training set, GSE14520 as the testing set, and ICGC-LIRI as the external validation set. We applied a 10-fold cross-validation method, utilizing 101 machine learning algorithms, including stepwise Cox regression, Lasso regression, Ridge regression, Partial Least Squares regression (plsRcox), CoxBoost, Random Survival Forest (RSF), Generalized Boosted Regression Modeling (GBM), Elastic Net (Enet), Supervised Principal Component Analysis (SuperPC), and Survival Support Vector Machine (survival-SVM) to construct the model.

#### 2.7.3 Model evaluation and selection

All constructed models were evaluated in the GSE14520 testing set and ICGC-LIRI external validation set. For each model, we calculated the concordance index (C-index) and assessed its performance in the training, testing, and external validation sets. The model with the highest C-index was selected as the final prognostic model, named the Polyamine metabolism-related signature (PMRS). Based on the median riskscore of the selected model, we divided the samples into high-risk and low-risk groups.

To further evaluate the predictive ability of the model, we performed kaplan-meier analysis to compare significant differences in overall survival (OS), disease-specific survival (DSS), disease-free survival (DFS), and progression-free survival (PFS) between high-risk and low-risk groups (log-rank test, P < 0.05). Additionally, we used Receiver Operating Characteristic (ROC) curves to assess the model’s accuracy.

### 2.8 Cell-cell communication analysis

We performed cell-cell interaction analysis using the “CellChat” R package (21). By default, the CellChatDB ligand-receptor database was used, following the standard CellChat analysis protocol. We inferred the specific interaction patterns between tumor cells and various cell types by detecting the expression of feature genes within the tumor cells. This approach enabled us to uncover potential communication networks between tumor cells and the surrounding immune and stromal cells, providing insights into how cell-cell signaling might influence tumor progression and the tumor microenvironment (TME).

### 2.9 Clinical feature analysis and prognostic nomogram construction and evaluation

We explored the correlation between risk scores and various clinical features, including age, gender, status, recurrence, grade, T stage, non-fibrotic alcoholic liver disease (NFALD), hepatitis B virus (HBV), hepatitis C virus (HCV), stage, and vascular invasion. Additionally, univariate and multivariate Cox regression analyses were performed on the TCGA-LIHC, GSE14520, and ICGC-LIRI datasets to assess whether the riskscore serves as an independent prognostic factor for HCC patients. Subsequently, we constructed a prognostic nomogram using the “rms” and “replot” R packages. The predictive performance of the nomogram was evaluated through ROC curves, calibration curves, and decision curve analysis (DCA) (22). These evaluations helped assess the nomogram’s ability to accurately predict overall survival and guide clinical decision-making, providing a valuable tool for individualized prognosis prediction in HCC.

### 2.10 Mutation landscape and copy number variation analysis

We obtained somatic mutation data for HCC samples from the TCGA database, stored in MAF (Mutation Annotation Format) files. Using the R package “maftools,” we visualized the top 20 mutated genes in the high-risk group.

Mutant-Allele Tumor Heterogeneity (MATH) is a method used to quantify variation in mutation allele frequencies within a tumor, reflecting genetic heterogeneity. The MATH score indicates the degree of variation in mutated alleles: higher MATH scores correspond to greater genetic heterogeneity and a higher mutation burden (23, 24). We calculated the MATH scores for HCC based on the distribution of mutated allele frequencies at tumor-specific mutation sites. Next, we performed copy number variation (CNV) analysis on the top 20 genes exhibiting the most significant CNV differences between the high- and low-risk groups. This CNV analysis provides valuable insights into the genetic alterations that may drive HCC progression and contribute to tumor heterogeneity, aiding in the identification of potential therapeutic targets for precision medicine in HCC treatment.

### 2.11 Tumor immune microenvironment analysis

To investigate the impact of different risk features on the tumor immune microenvironment (TIME) of HCC, we first assessed the immune response to HCC immunotherapy using the Tumor Immune Dysfunction and Exclusion (TIDE) analysis (http://tide.dfci.harvard.edu/). TIDE is a computational method designed to predict tumor response to immunotherapy based on gene expression profiles. We then employed the R package “ESTIMATE” to evaluate the stromal score, immune score, estimate score and tumor purity for each patient. The ESTIMATE algorithm calculates these scores based on the expression of genes in the stromal and immune components of the TME, helping to assess the overall immune landscape of the tumor. To gain deeper insights into the TIME, we applied the R package “IOBR” (https://github.com/IOBR/IOBR), which integrates several deconvolution methods and signature construction tools. The IOBR package includes 8 immune infiltration analysis algorithms, such as XCELL, TIMER, QUANTISEQ, MCPCOUNTER, EPIC, CIBERSORT-ABS, CIBERSORT and IPS, enabling a comprehensive evaluation of immune cell infiltration patterns and TME composition (25). Additionally, we utilized the CIBERSORT algorithm to compare immune cell infiltration profiles between different risk subgroups. This method estimates the relative abundances of various immune cell types based on gene expression data, offering insights into how risk stratification influences the immune landscape of HCC. Finally, we retrieved immune-related pathway gene sets from the KEGG database (https://www.genome.jp/kegg/) (**Supplementary Table S7**). These pathways provide valuable information on the molecular mechanisms by which immune cells interact with the tumor and contribute to HCC progression or immune evasion.

### 2.12 Drug sensitivity analysis

To explore the relationship between different risk features and drug sensitivity in HCC, we utilized the “oncoPredict” R package, which compares drug IC50 values from the GDSC (Genomics of Drug Sensitivity in Cancer) database. OncoPredict is designed to predict cancer patients’ sensitivity to various drugs, assisting researchers and clinicians in tailoring personalized treatment strategies (26).Additionally, we obtained drug sensitivity data from the CTRP (Cancer Therapeutics Response Portal) database (https://portals.broadinstitute.org/ctrp.v2.1/) and the PRISM (PharmacoGenomics of Cancer Cell Lines) database (https://www.theprismlab.org/), which provide drug response profiles for cancer cell lines (CCLs) (27–29). These databases offer valuable insights into the drug response profiles of a wide range of cancer cell lines, helping to predict patient-specific drug sensitivity. We also collected transcriptomic data from the CCLE (Cancer Cell Line Encyclopedia) database and applied a ridge regression model to generate drug sensitivity estimates for each patient. Both the CTRP and PRISM databases provide the area under the dose-response curve (AUC) as a standard measure of drug sensitivity. A lower AUC value indicates higher drug sensitivity, while a higher AUC suggests lower drug sensitivity. In the last, we analyzed the relationship between riskscore and the IC50 or AUC values to identify potential drugs that may be effective in treating HCC. This analysis aids in understanding which drugs are likely to be most effective for patients with different risk profiles and can inform future therapeutic strategies.

### 2.13 Quantitative real-time polymerase chain reaction

Total RNA was extracted from different HCC cell lines using Trizol reagent (Takara, Japan). The RNA was then reverse transcribed into complementary DNA (cDNA) using the PrimeScript RT reagent kit (Takara, Japan) according to the manufacturer’s instructions. Gene expression analysis was performed by RT-qPCR using TB Green Premix Ex Taq (Takara, Japan). PCR primers were synthesized by Tsingke (Beijing, China), and primer details are provided in **Supplementary Table S8**. The PCR conditions were as follows: initial denaturation at 95°C for 5 minutes, followed by 40 cycles of the three-step PCR process (95°C for 40 seconds, 60°C for 50 seconds, and 72°C for 30 seconds). The results were analyzed using the comparative Ct method, with the Ct values of each gene normalized to the corresponding GAPDH Ct values. Data are presented as the mean ± standard deviation (SD) of three independent experiments. Gene expression levels were quantitatively analyzed using the 2-ΔΔCT method.

### 2.14 Statistical analysis

All statistical analyses were performed using R software (version 4.3.2). Continuous variables between two groups were compared using either the Wilcoxon test or t-test, while categorical variables were assessed using the χ² test or Fisher’s exact test. The correlation between two continuous variables was determined using spearman’s correlation analysis. OS was compared using kaplan-meier survival analysis and the log-rank test. All p-values were calculated using a two-tailed test and adjusted for multiple comparisons using the FDR method. A p-value of < 0.05 was considered statistically significant. This statistical approach ensures the robustness of the results and helps mitigate potential biases or errors arising from multiple comparisons in high-dimensional data analysis.

## 3 Results

### 3.1 Consensus clustering analysis

**Figure 1:**
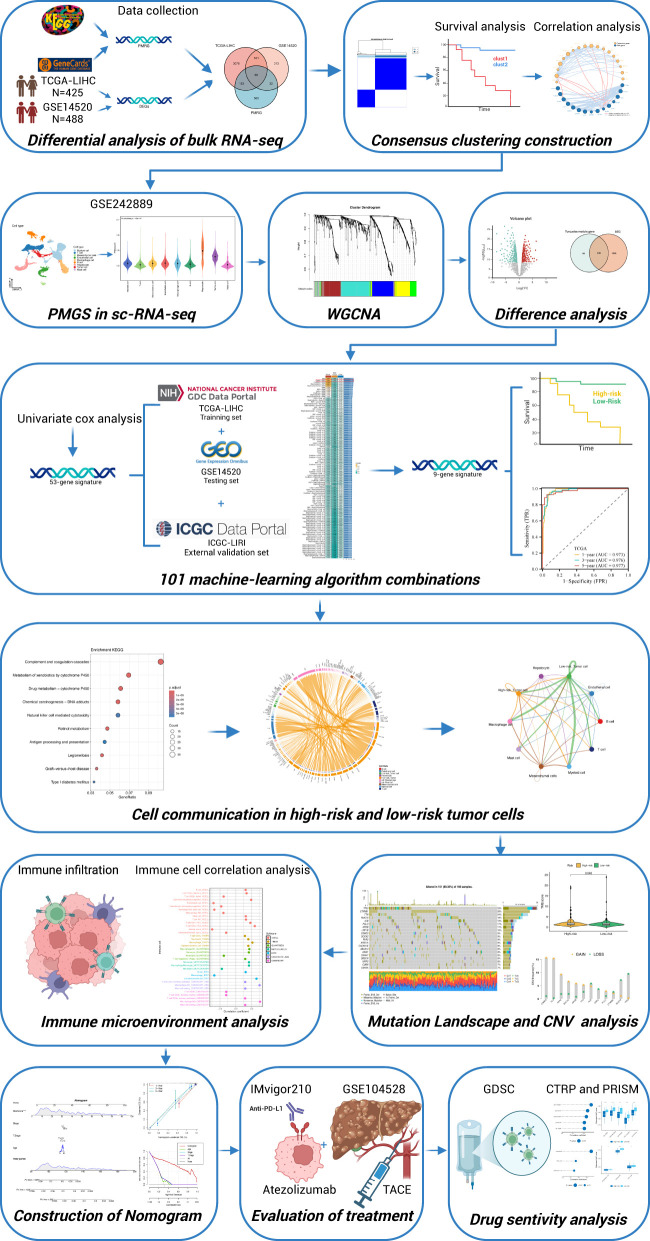
Flowchart of this study.

**Figure 2:**
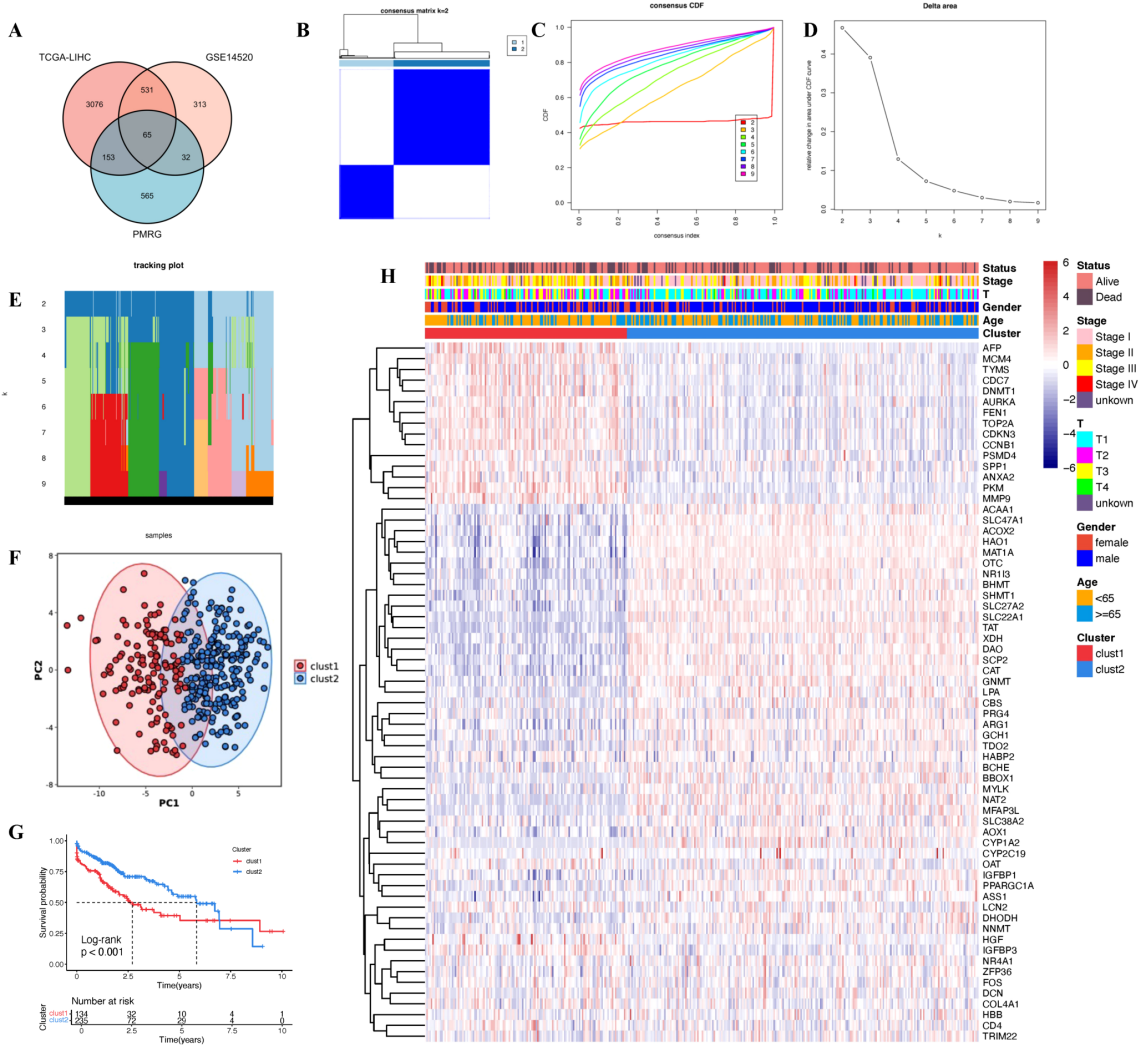
Consensus clustering construction. **(A)** Venn plot showing the intersecting genes between PMRG and DEGs in bulk RNA-seq. **(B)** Consistency matrix heatmap. **(C)** Cumulative distribution function. **(D)** Delta area plot. **(E)** Tracking plot. **(F)** PCA plot. **(G)** Kaplan-Meier survival analysis. **(H)** Clinical feature heatmap.

The flowchart of this study is depicted in **Figure 1**. Initially, we performed differential expression analysis between normal and tumor samples in the TCGA-LIHC and GSE14520 cohorts, with selection criteria of adjusted p-values < 0.05 and |log2 fold change| > 1. Among the 814 genes related to polyamine metabolism, 65 differentially expressed polyamine metabolism-related genes(PMRG) were identified through intersection filtering (**Figure 2A**). Next, we conducted unsupervised consensus clustering analysis on these 65 differentially expressed PMRG. The results indicated that the optimal clustering was achieved when K = 2, dividing the samples into two groups: polyamine metabolism-enriched (clust1) and polyamine metabolism-deficient (clust2) (**Figures 2B–E**). Principal Component Analysis (PCA) demonstrated a clear distribution difference between clust1 and clust2 (**Figure 2F**). Kaplan-Meier survival analysis revealed that patients in the clust1 group had a significantly poorer prognosis (**Figure 2G**). A heatmap further validated the consistency of the clustering results with the differential expression analysis (**Figure 2H**). This approach effectively identifies distinct polyamine metabolism-related subtypes in HCC, which could have important implications for prognosis and therapeutic strategies.

### 3.2 TIME of different polyamine metabolism subtypes

**Figure 3:**
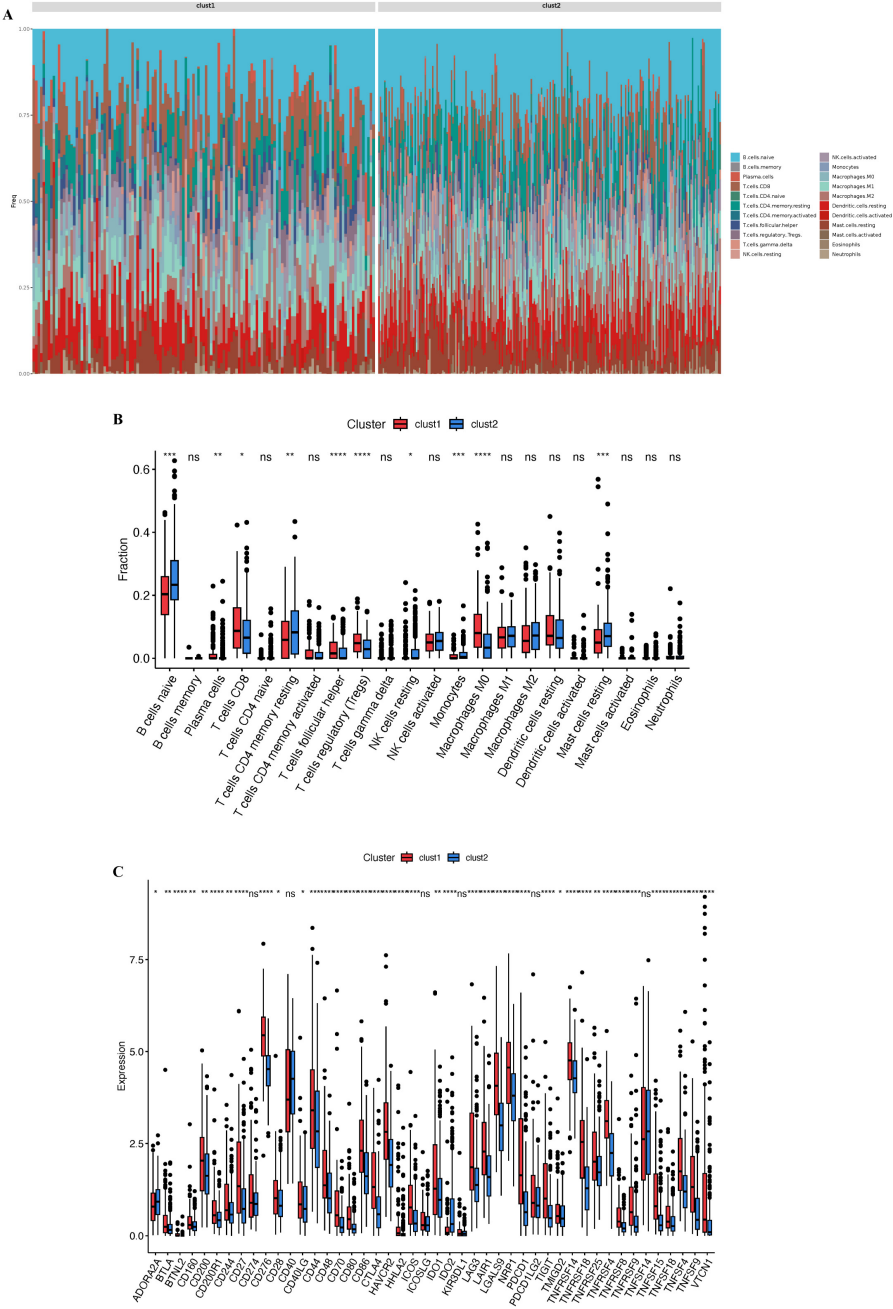
Assessment of immune cell infiltration and checkpoint in HCC. **(A)** Composition and relative abundance of 22 immune cell types in TCGA-LIHC samples. **(B)** Box plot illustrating the differential analysis of immune cell infiltration associated with clust1 and clust2. **(C)** Differences in the expression of immune checkpoint genes between the clust1 and clust2. NS, not statistically significant; *p < 0.05;**p < 0.01; ***p < 0.001;****p < 0.0001.

To further quantify the differences in immune cell infiltration between the clust1 and clust2 subtypes, we utilized the CIBERSORT algorithm to assess immune cell infiltration abundance in each sample (**Figure 3A**). The results indicated that the clust1 subtype exhibited higher abundance of immune cells with antitumor functions, including Plasma cells, T cells CD8, T cells follicular helper, T cells regulatory (Tregs), and Macrophages M0. In contrast, the clust2 subtype was characterized by a higher prevalence of cell types with weaker anticancer activity, such as naive B cells naive, T cells CD4 memory resting, NK cells resting, Monocytes, and Mast cells resting (**Figure 3B**). Additionally, we analyzed the expression of 46 immune checkpoint genes and found significant differences in expression between the two subtypes, except for CD274, CD40, ICOSLG, KIR3DL1, PDCD1LG2, and TNFSF14. Specifically, ADORA2A and IDO2 showed lower expression in clust1, while the other 37 immune checkpoints were expressed at higher levels in clust1 (**Figure 3C**).

To evaluate the correlation between immune escape and immune response, we employed the Tumor Immune Dysfunction and Exclusion (TIDE) algorithm (http://tide.dfci.harvard.edu/). The results revealed that clust1 had higher expression levels of IFNG, Exclusion, MDSC, and TIDE, indicating a higher proportion of non-responders to immunotherapy in this subtype. Conversely, clust2 displayed higher Dysfunction scores (**Supplementary Figures S1A–F**). The ImmuneScore, calculated using the ESTIMATE algorithm, showed that the immuneScore of clust1 was significantly higher than that of clust2 (**Supplementary Figure S1G**). Furthermore, the immunotherapy response Score (IPS), which predicts responses to CTLA-4 and PD-1 inhibitors, was higher in clust1 patients under both ips_ctla4_neg_pd1_neg (CTLA4-/PD1-) and ips_ctla4_pos_pd1_neg (CTLA4+/PD1-) treatment conditions (**Supplementary Figures S4H, I**). These findings suggest that the clust1 subtype may exhibit stronger immune activity or anticancer potential.

In summary, the different polyamine metabolism subtypes exhibit significant variations in the tumor immune microenvironment. The clust1 subtype shows higher immune activity, while the clust2 subtype may be associated with immune suppression. These results highlight the importance of stratifying HCC patients based on polyamine metabolism features to better understand their TIME, which could inform potential therapeutic strategies.

### 3.3 Differentially expressed genes and functional enrichment analysis in different polyamine metabolism subtypes

**Figure 4:**
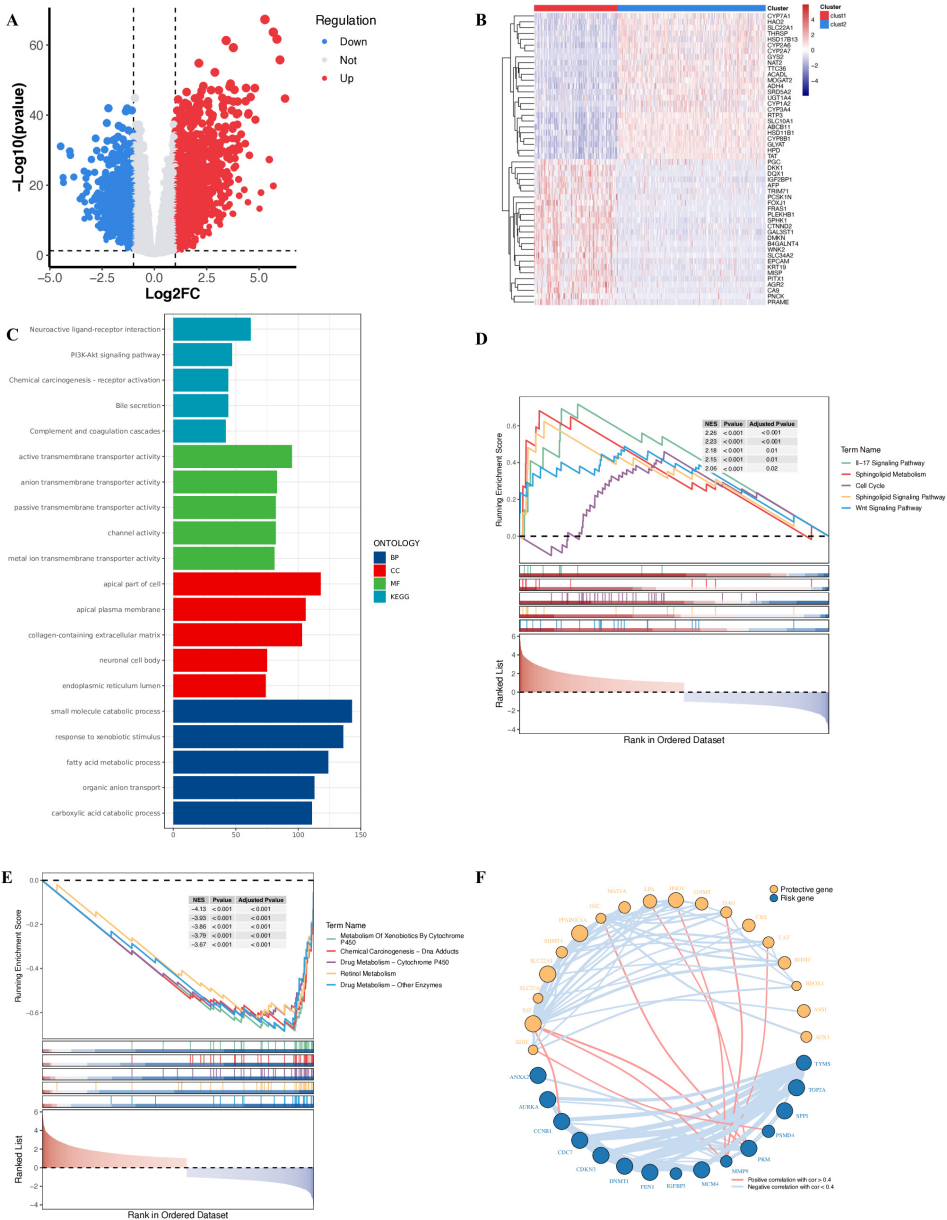
Differential expression and enrichment analysis between the clust1 and clust2 subtypes. **(A)** Volcano plot of differentially expressed genes between the clust1 and clust2 subtypes. **(B)** The top 50 differentially expressed genes in the clust1 and clust2 subtypes. **(C)** A bar chart displaying the functional enrichment analysis outcomes for both the clust1 and clust2 subtypes. **(D, E)** GSEA enrichment analysis based on KEGG pathways. **(F)** The results of the univariate cox regression analysis of PMRG and the correlations among 65 genes.

Using the “limma” package, we performed differential expression analysis between the two polyamine metabolism subtypes and identified 2,242 differentially expressed genes (DEGs) associated with polyamine metabolism (**Supplementary Table S9**). The volcano plot of these DEGs and the heatmap of the top 50 genes are shown in **Figures 4A, B**, respectively. Next, we conducted Kyoto Encyclopedia of Genes and Genomes (KEGG) pathway analysis and Gene Ontology (GO) enrichment analysis. The results revealed that the DEGs between clust1 and clust2 were significantly enriched in pathways related to “Neuroactive ligand-receptor interaction” and “Small molecule catabolic process” (**Figure 4C**). In the Gene Set Enrichment Analysis (GSEA) based on KEGG gene sets, we found that the highly expressed genes were significantly enriched in the “IL-17 Signaling Pathway” (**Figure 4D**), while the lowly expressed genes were significantly enriched in the “Metabolism of Xenobiotics by Cytochrome P450” pathway (**Figure 4E**). Finally, we performed univariate Cox regression analysis and correlation analysis on the 65 polyamine metabolism-related genes, resulting in the identification of 33 genes for further analysis (**Figure 4F**, **Supplementary Figure S1J**).

### 3.4 Polyamine metabolic features in single-cell transcriptome

To further investigate the polyamine metabolic features in the single-cell transcriptome, we downloaded scRNA-seq data (GSE242889) from the GEO database. After performing quality control, the violin plots of the data are shown in **Supplementary Figures S2A–D**. We then log-transformed and normalized the data, identifying the top 2,000 most variable genes. Dimensionality reduction was performed using PCA and Uniform Manifold Approximation and Projection (UMAP), resulting in a reduced dataset of 37,330 cells.

**Figure 5:**
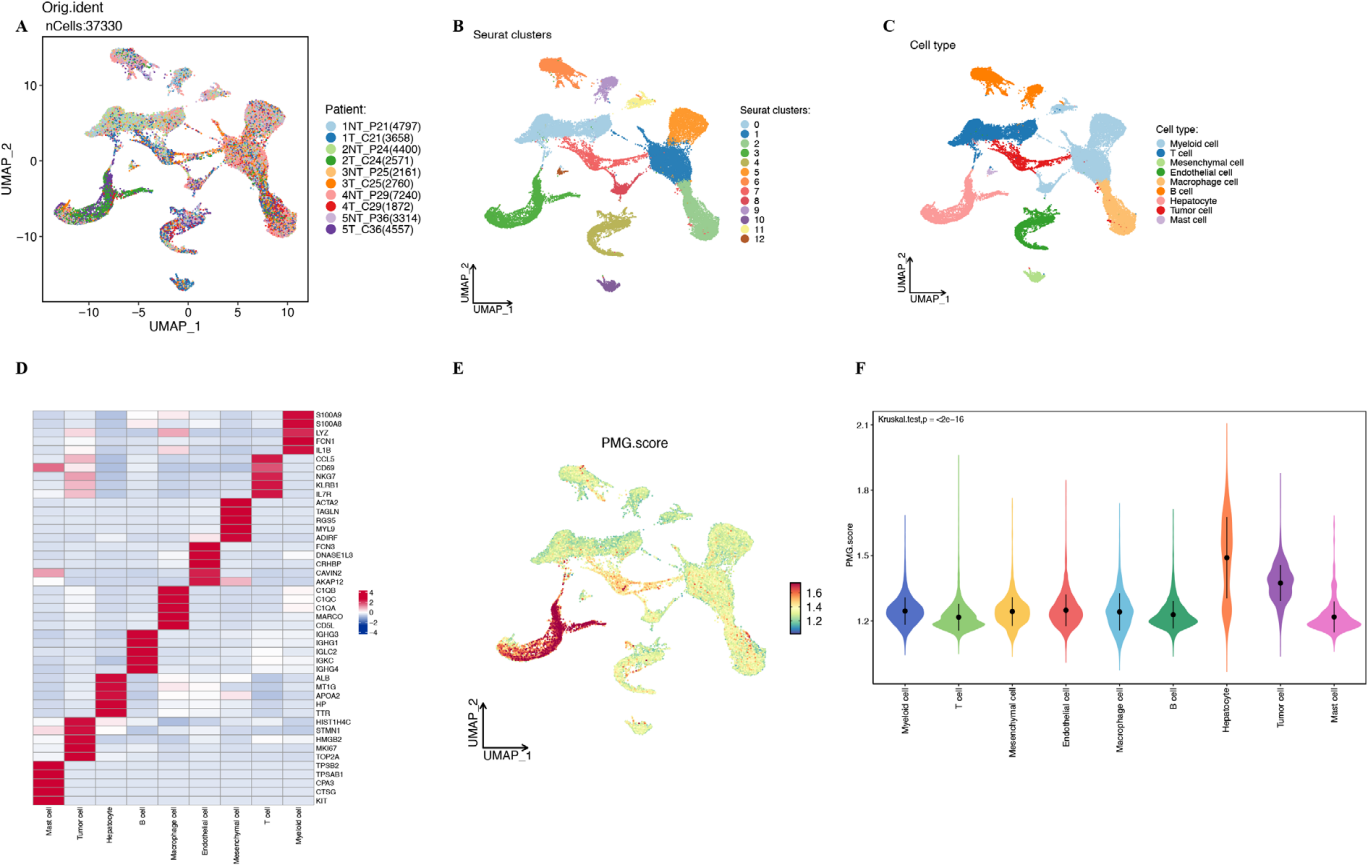
Polyamine metabolism characteristic in the single cell transcriptome. **(A)** The UMAP plot of ten samples from the GSE242889 dataset, colored to indicate the sample names. **(B, C)** The results of cell clustering and annotation for the GSE242889 dataset. **(D)** Heatmap showing the top 5 marker genes in each cell cluster. **(E)** The activity score of PMG score in each cell. **(F)** The distribution of the PMG score in different cell types.

To address batch effects, we conducted CCA, with the batch-corrected results displayed in **Figure 5A**. Clustering analysis (resolution = 0.1) revealed 13 distinct clusters (**Figure 5B**). The annotation of the cell clusters was as follows: Cluster 0 as T cell, Clusters 1, 5, 8, and 11 as myeloid cell, Cluster 2 as macrophage cell, Cluster 3 as hepatocyte, Cluster 4 as endothelial cell, Clusters 6 and 9 as B cell, Cluster 7 as tumor cell, Cluster 10 as mesenchymal cell, and Cluster 12 as mast cell. The annotated UMAP plot is shown in **Figure 5C**. Using the “FindAllMarkers” function in the Seurat package, we identified the marker genes for each cluster, applying thresholds of log2FC > 0.25 and minimum percentage of cells (min.pct) > 0.25. The heatmap of the top 5 marker genes for each cluster is presented in **Figure 5D**. To quantify the polyamine metabolic gene (PMG) features in each cell type, we used the “AddModuleScore” function in Seurat to calculate the expression levels of a gene set consisting of 33 polyamine metabolism-related genes (**Figure 5E**). The results revealed significant differences in polyamine metabolic features between normal and tumor cells (**Figure 5F**). Based on these PMG features, we classified the cells into high PMGscore(PMGS) and low PMGscore(PMGS) groups and identified 915 differentially expressed genes (**Supplementary Table S10**).

### 3.5 WGCNA analysis and identification of core modules related to PMGS

**Figure 6:**
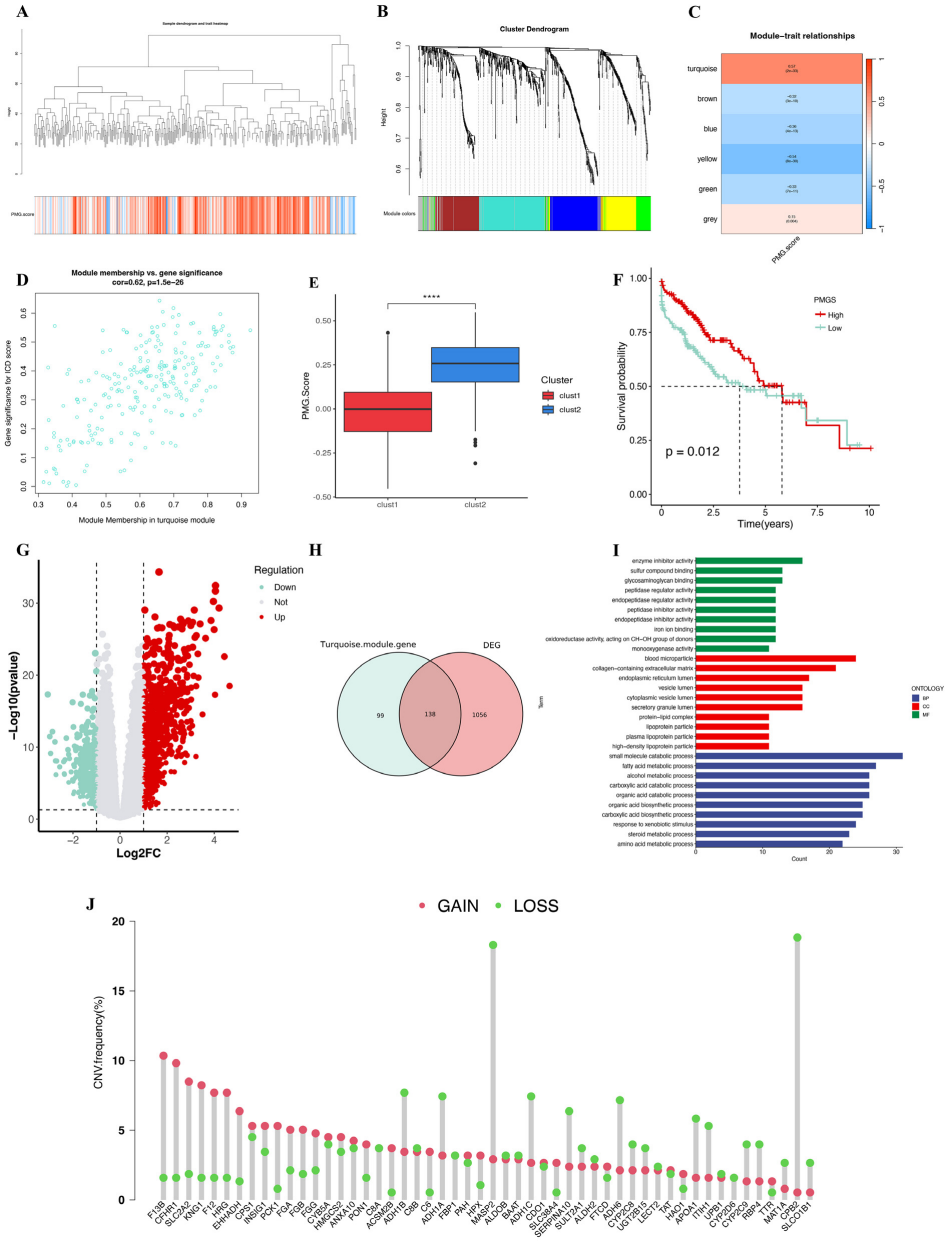
Identification of the immunogenic polyamine metabolism-related genes (PMRG). **(A)** Dendrogram showing the hierarchical clustering of TCGA-LIHC samples. The bottom heatmap represents each sample’s PMG score, calculated by ssGSEA algorithm. **(B)** Cluster dendrogram of the WGCNA analysis. **(C)** Module-trait heatmap showing that the turquoise module was closely related to the polyamine metabolism trait. **(D)** Scatter plot showing the relationship between gene significance (GS) and module membership (MM) in the turquoise module. **(E)** The box plot displays the difference in PMG score between the clust1 and clust2 subtypes. **(F)** Kaplan-Meier survival analysis of overall survival (OS) in the TCGA-LIHC cohort, comparing high and low PMG scores (PMGS). **(G)** A volcano plot showing the results of differential analysis between high PMG score (PMGS) and low PMG score (PMGS) samples in the TCGA-LIHC cohort. **(H)** A Venn diagram illustrating the overlapping genes between the turquoise module and DEGs associated with high PMGS, as well as those associated with low PMGS in bulk RNA-seq analysis. **(I)** GO enrichment of the overlapping genes. **(J)** Copy number variation (CNV) frequency of PMRG presented in **Supplementary Table S13**.

To explore genes associated with polyamine metabolism, we quantified the PMGS for each TCGA-LIHC sample using the single-sample gene ssGSEA method. To identify modules significantly correlated with the PMGS, we constructed a WGCNA based on differentially expressed PMRG at the single-cell level (**Figure 6A**). The optimal soft threshold was selected as 5 (R² = 0.895), ensuring that the network adhered to the scale-free topology criterion (**Supplementary Figure S2E**). The minimum number of genes per module was set to 50, and the module dendrogram was cut at a MEDissThres of 0.15, resulting in the identification of six distinct modules (**Figure 6B**). Among these, the turquoise module showed a strong correlation with the PMG score in TCGA-LIHC (**Figure 6C**). The scatter plot in **Figure 6D** demonstrates the correlation between gene significance (GS) and module membership (MM) within the turquoise module.In summary, the genes within the turquoise module may play a key role in the functional pathways related to polyamine metabolism (**Supplementary Table S11**).

### 3.6 Differential analysis between high and low PMGS

There was a significant difference in PMGS between the clust1 and clust2 subtypes (**Figure 6E**, P < 0.0001). Based on the median PMGS in TCGA-LIHC, the samples were divided into high and low PMGS groups. Kaplan-Meier survival analysis demonstrated that HCC patients with high PMGS had a significantly better prognosis compared to those with low PMGS (**Figure 6F**). Subsequently, we conducted differential expression analysis using the “limma” package between the high and low PMGS groups, identifying 1,194 differentially expressed genes (DEGs) (**Supplementary Table S12**). Of these, 755 genes were upregulated, while 439 genes were downregulated (**Figure 6G**).

To further investigate genes related to polyamine metabolism, we performed an intersection analysis between the DEGs and the genes in the turquoise module, which identified 138 PMRG (**Figure 6H**). Gene Ontology (GO) enrichment analysis of these 138 genes revealed significant associations with BP, CC, and MF, particularly in processes such as small molecule catabolic processes, blood microparticles, and enzyme inhibitor activity (**Figure 6I**).

We then performed univariate Cox regression analysis on these 138 genes and selected 53 genes for further analysis and machine learning model construction (**Supplementary Table S13**). Additionally, we analyzed the copy number variations (CNVs) of these 53 genes and found that the CNV frequencies of MASP2 and CPB2 were increased by more than 15% (**Figure 6J**). Finally, the protein-protein interaction (PPI) network of these 53 genes is shown in **Supplementary Figure S2G**.

### 3.7 Development of a prognostic gene signature for HCC patients based on machine learning

**Figure 7:**
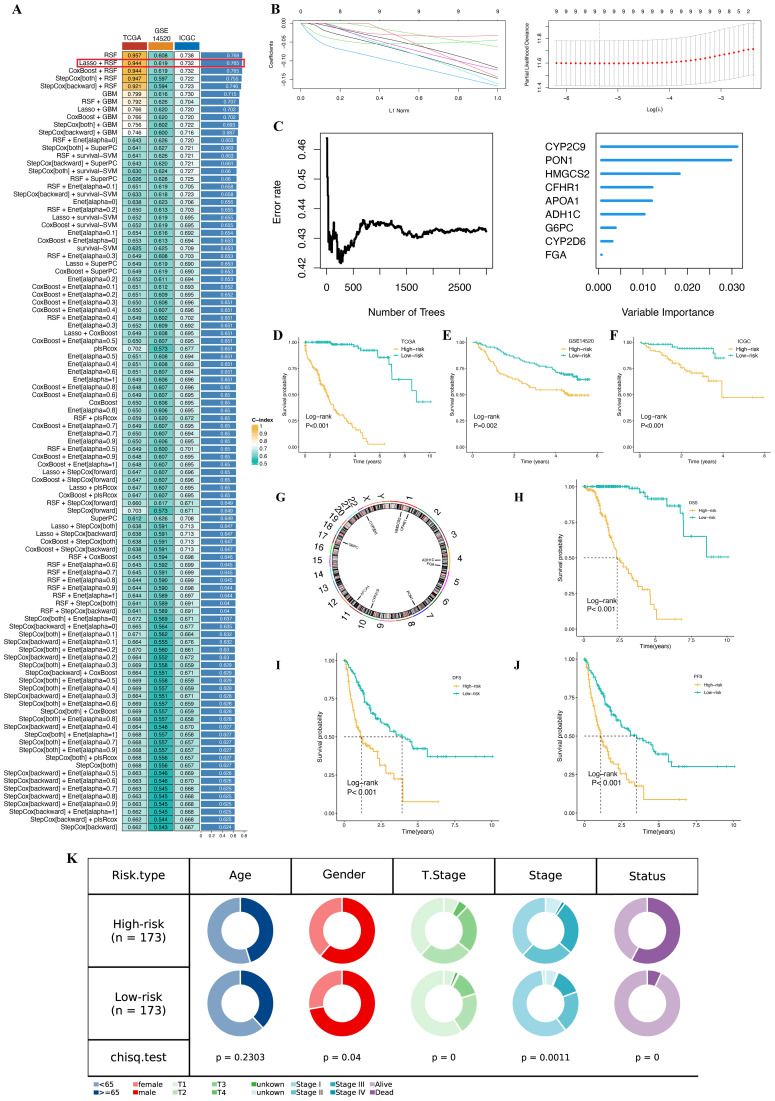
Machine learning and PMRS model development and validation. **(A)**. A total of 101 kinds of prediction models via a tenfold cross-validation framework and further calculated the C index of each model across all validation datasets. **(B)** Visualization of LASSO regression in the TCGA-LIHC cohort. **(C)** Analysis of the number of trees required to achieve minimal error in the model and the significance of the nine genes using the Random Survival Forest (RSF) algorithm. **(D, F)** Kaplan-Meier survival curves showing overall survival (OS) based on the PMRS in the TCGA training set, GSE14520 testing set, and ICGC external validation set. **(G)** Chromosomal distribution of the nine genes included in the PMRS. **(H–J)** Kaplan–Meier survival curves for DSS, DFS, and PFS based on the PMRS in the TCGA-LIHC cohort. **(K)** Pie plot of the difference in clinical characteristics between high- and low-risk groups.

To develop a polyamine metabolism-related signature (PMRS), we applied a comprehensive analysis using 101 machine learning algorithms, selecting 53 prognostic genes identified through univariate Cox regression analysis. TCGA-LIHC data was used as the training set, GSE14520 data as the testing set, and ICGC-LIRI as the external validation set. We evaluated the consistency index (C-index) across all datasets (training, testing, and validation) using a 10-fold cross-validation approach (**Figure 7A**). Among the models assessed, the top five, ranked by the average C-index, were all developed using the Random Survival Forest (RSF) algorithm. However, as the RSF model showed a lower C-index in the testing set compared to the Lasso+RSF model, we selected the Lasso+RSF model as the most accurate and clinically relevant prediction model. Using this model, we developed a clinical prognostic risk score model (PMRS) that includes 9 genes: CYP2C9, PON1, HMGCS2, CFHR1, APOA1, ADH1C, G6PC, CYP2D6, and FGA (**Figures 7B, C**, **Supplementary Table S14**). Samples were divided into high-risk and low-risk groups based on the median risk score. Kaplan-Meier survival analysis showed that the high-risk group had a significantly poorer prognosis compared to the low-risk group (**Figure 7D**). Similar results were observed in both the testing set and external validation set (**Figures 7E–F**). The chromosomal locations of the 9 genes are shown in **Figure 7G**. In the TCGA-LIHC cohort, patients in the high-risk group exhibited significantly poorer DSS, DFS, and PFS compared to the low-risk group (**Figures 7H–J**). Moreover, we compared the clinical and pathological characteristics of patients in the high-risk and low-risk groups and found significant differences in gender, T stage, stage, and status (P < 0.05; **Figure 7K**).

### 3.8 PMRS model evaluation

**Figure 8:**
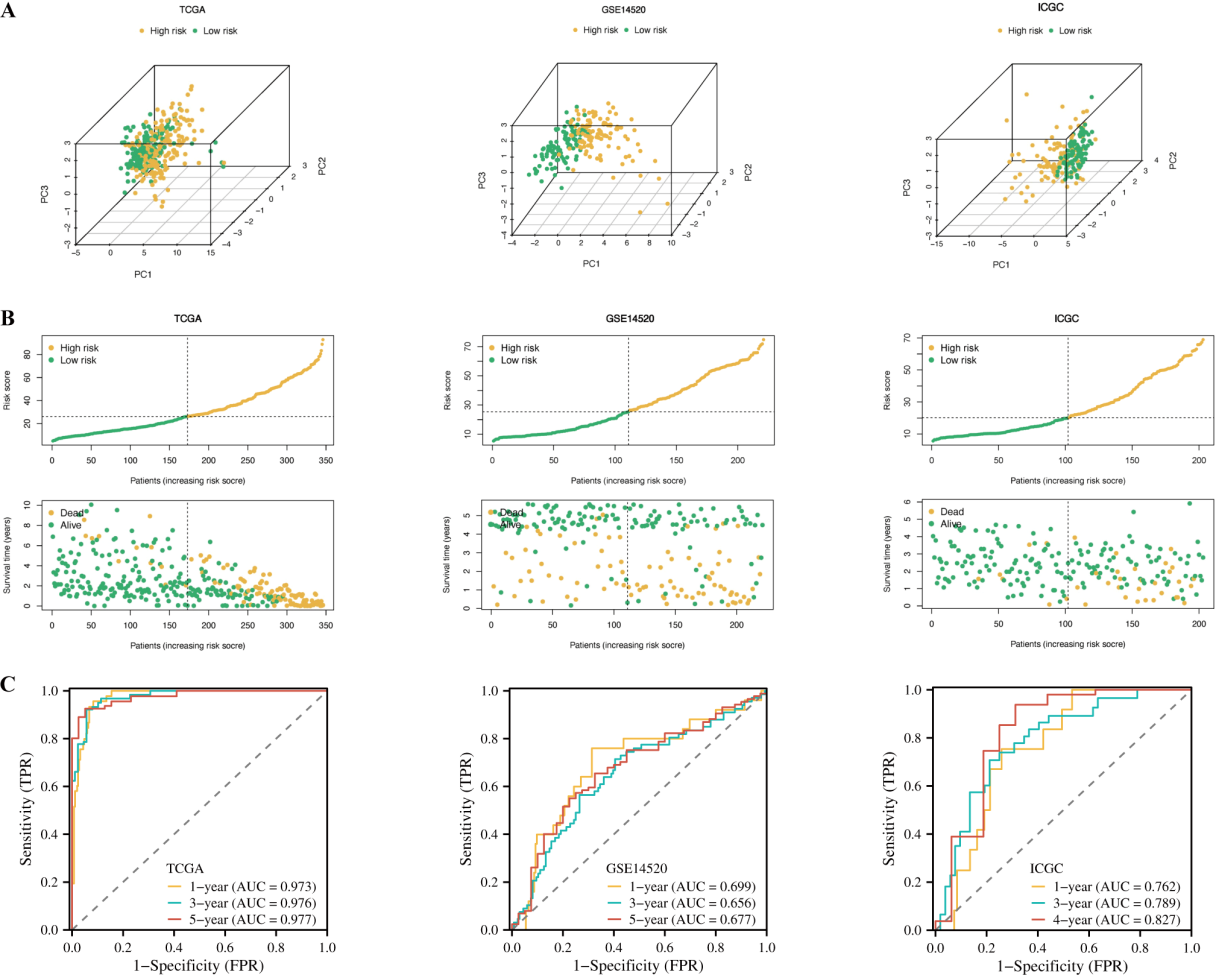
Evaluation of the PMRS model. **(A)** Principal component analysis (PCA) plot based on the PMRS in the TCGA, GSE14520, and ICGC cohorts. **(B)** Distribution of PMRS according to survival status and time in the TCGA, GSE14520, and ICGC cohorts. **(C)** Evaluating the predictive accuracy of the PMRS for OS in the TCGA-LIHC, GSE14520, and ICGC cohorts using ROC curves.

To evaluate the predictive performance of the PMRS model, we conducted PCA across three cohorts to confirm the separation between high-risk and low-risk groups (**Figure 8A**). The distribution of riskscores and the survival status plot revealed that the high-risk group had higher riskscores and a greater proportion of deceased patients (**Figure 8B**). The area under the receiver operating characteristic (ROC) curve (AUC) was calculated for different survival time points. In the TCGA training set, the AUC values were 0.973 for 1-year, 0.976 for 3-year, and 0.977 for 5-year survival. In the GSE14520 testing set, the AUC values were 0.699 for 1-year, 0.656 for 3-year, and 0.677 for 5-year survival. For the ICGC-LIRI external validation set, there were no deaths recorded after the fifth year, resulting in a 5-year AUC of 0. The AUC values for 1-year, 3-year, and 4-year survival intervals in this cohort were 0.762, 0.789, and 0.827, respectively (**Figure 8C**). These results demonstrate that the PMRS model provides robust predictive performance, particularly for 1-year, 3-year, and 4-year survival, where the model shows statistically significant findings. The predictive accuracy is well-supported by sufficient event data across these time intervals.

### 3.9 Correlation of PMRS with single-cell characteristics

**Figure 9:**
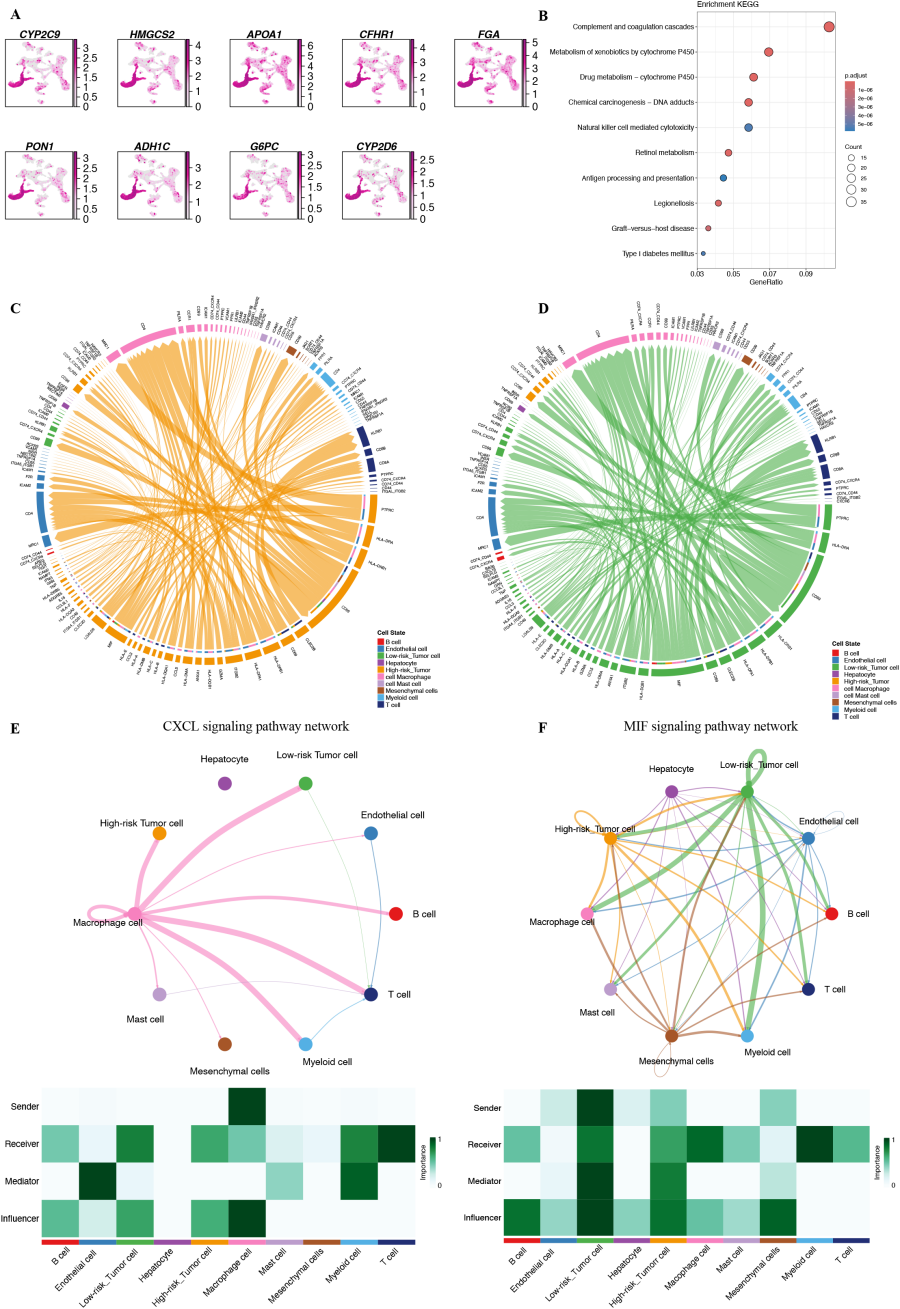
The correlation of PMRS with single-cell characteristics. **(A)** Expression of CYP2C9, HMGCS2, APOA1, CFHR1, FGA, PON1, ADH1C, G6PC, and CYP2D6 across various cell types as determined by single-cell RNA-seq analysis. **(B)** KEGG analysis of the DEGs between the high and low-risk cells. **(C, D)** The ligand-receptor interactions sent from high-risk tumor cells and low-risk tumor cells. **(E, F)** Circos plots illustrating the CXCL and MIF signaling pathway networks, along with heatmaps depicting the involvement of different cell types in these pathway networks.

To explore the impact of different risk features on the TME at the single-cell transcriptomic level, we analyzed the expression of nine genes across various cell types (**Figure 9A**). The results indicated that these genes were most highly expressed in hepatocyte, followed by immune cells, with the lowest expression observed in tumor cell.

Based on the expression levels of these nine genes, we calculated a riskscore for each cell and identified differentially expressed genes between high-risk and low-risk groups. KEGG enrichment analysis revealed that the differentially expressed genes were significantly enriched in several pathways, including: Complement and coagulation cascades, Metabolism of xenobiotics by cytochrome P450, Drug metabolism − cytochrome P450, Chemical carcinogenesis − DNA adducts, and Natural killer cell-mediated cytotoxicity (**Figure 9B**). we calculated the riskscore for each cell based on the PMRS model, classifying tumor cell into high-risk and low-risk groups. We then examined their interactions with other immune cells. The results showed significant differences in the communication patterns between tumor cells with different risk features (**Figures 9C, D**). In the TME, various cell types function as senders, receivers, signal mediators, and regulators, facilitating the transmission of intercellular signals. Tumor cell in the low-risk group communicated with a broader range of immune cells and exhibited stronger inbound and outbound signaling in the CXCL and MIF signaling pathway networks. These cells acted as more potent mediators and influencers (**Figures 9E, F**). In summary, tumor cells with low-risk scores engage in complex signaling exchanges with immune cells through the CXCL and MIF pathways, potentially contributing to immune escape and promoting tumor progression. The clinical significance of this phenomenon in tumor immunotherapy warrants further investigation.

### 3.10 Comparison of clinical and pathological characteristics of patients with PMRS and construction of nomogram

We examined the relationship between clinical and pathological characteristics and various risk features in HCC patients. The results revealed that higher risk scores were strongly associated with several clinical features, including gender, status, recurrence, grade, T stage, HBV, stage, and vascular invasion (**Supplementary Figures S3A–K**). **Supplementary Figure S3L** illustrates the correlation between the expression levels of the nine genes and these clinical characteristics. Additionally, in the GSE14520 cohort, riskscore significantly differed with Predicted risk Metastasis Signature, such as Main Tumor Size, Multinodular, AFP levels, Stage, and BCLC stage (**Supplementary Figures S4A–J**).

**Figure 10:**
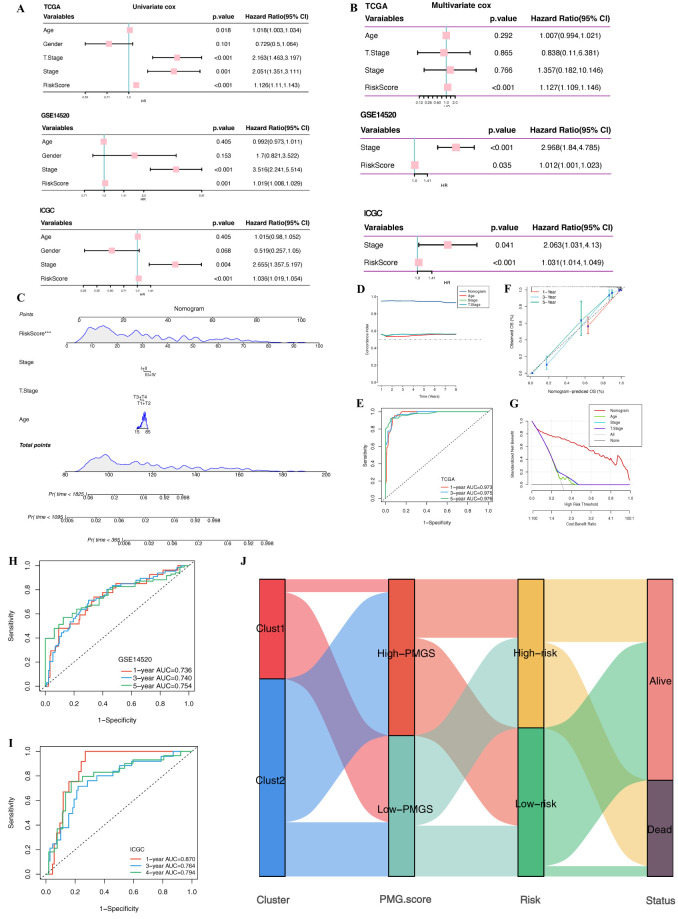
Construction and evaluation of the nomogram based on PMRS. **(A, B)** Univariate Cox and Multivariate Cox analysis of TCGA-LIHC,GSE14520 and ICGC cohorts. **(C)** Nomogram for predicting the 1-, 3-, and 5-year survival rates based on the PMRS. **(D)** The comparison of the C index between the nomogram and other clinical characteristics. **(E)** ROC curves illustrating the predictive performance of the nomogram for 1-, 3-, and 5-year OS in the TCGA-LIHC cohort. **(F)** Calibration curve of the nomogram for 1, 3, and 5-year OS. **(G)** Decision curve analysis (DCA) showing the net benefit by applying the nomogram and other clinical characteristics. **(H)** ROC curves illustrating the predictive performance of the nomogram for 1-, 3-, and 5-year OS in the GSE14520 cohort. **(I)** ROC curves illustrating the predictive performance of the nomogram for 1-, 3-, and 4-year OS in the ICGC cohort. **(J)** Alluvial diagram depicting the interrelationship between clust subtypes, PMGS, risk groups, and survival status in TCGA-LIHC patients.

To evaluate the potential of riskscore as an independent prognostic factor for HCC, we conducted univariate and multivariate Cox regression analyses using data from the TCGA-LIHC, GSE14520, and ICGC cohorts (**Figures 10A, B**). Univariate analysis showed that riskscore was a significant prognostic factor for HCC (HR > 1, P < 0.05), and multivariate analysis further confirmed the independence of riskscore as a prognostic factor (HR > 1, P < 0.05).

For clinical application, we constructed a nomogram incorporating riskscore and clinical characteristics (**Figure 10C**). In the TCGA-LIHC cohort, the nomogram demonstrated stable and robust predictive performance, particularly for OS predictions over 1 to 8 years. It outperformed other clinical features in terms of accuracy, with the AUC for 1-year, 3-year, and 5-year survival predictions being 0.973, 0.975, and 0.976, respectively. Calibration curve analysis showed a strong agreement between predicted and observed values. Additionally, decision curve analysis (DCA) revealed that the nomogram provided higher net clinical benefit compared to other clinical features (**Figures 10D–G**). In the GSE14520 cohort, the nomogram achieved AUCs of 0.736, 0.740, and 0.754 for 1-year, 3-year, and 5-year survival predictions, respectively (**Figure 10H**). In the ICGC-LIRI cohort, the AUCs for 1-year, 3-year, and 4-year survival predictions were 0.870, 0.764, and 0.794, respectively (**Figure 10I**), further validating the model’s predictive accuracy. These results suggest that the nomogram, developed using risk scores and clinical characteristics, is a reliable tool for personalized prognostic prediction in HCC patients. Furthermore, we used an alluvial diagram to illustrate the relationships between different polyamine metabolism subtypes, PMGS, and risk groups (**Figure 10J**). The results show that the clust1 subtype predominantly exhibits lower PMGS and higher risk score, correlating with a poorer prognosis.

### 3.11 Biological functions of PMRS

**Figure 11:**
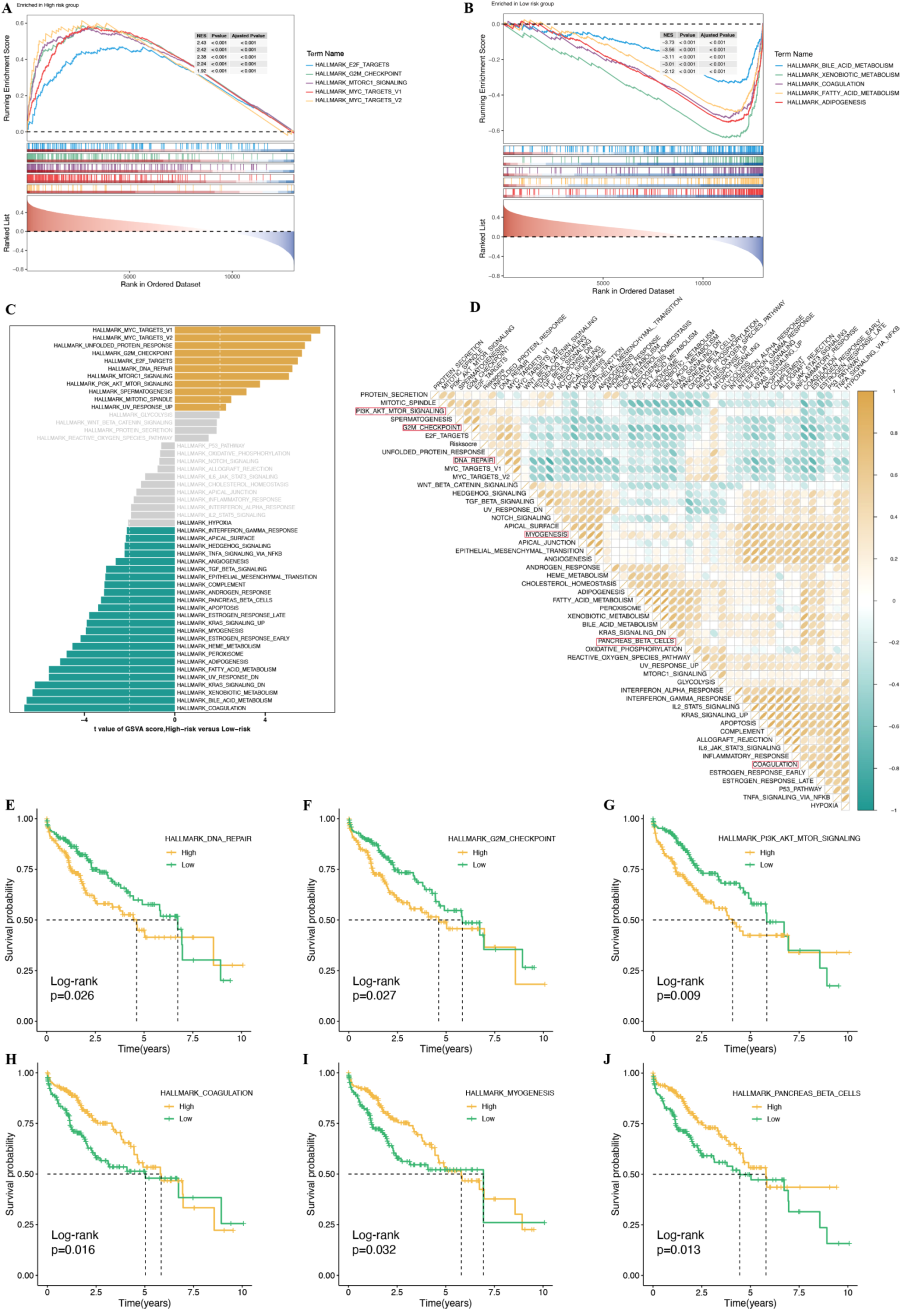
Transcriptome features of HCC patients with different PMRS. **(A, B)** GO terms enriched in the high-risk and low-risk groups based on GSEA analysis. **(C)** Differences in hallmark pathway activities between the high-risk and low-risk groups, as scored by GSVA. **(D)** Correlation between the risk score and hallmark pathway activities, as scored by GSVA. **(E–J)** Kaplan–Meier survival plots showing significant correlations between OS and GSVA scores for HALLMARK DNA REPAIR **(E)**, HALLMARK G2M CHECKPOINT **(F)**, HALLMARK PI3K AKT MTOR SIGNALING **(G)**, HALLMARK COAGULATION **(H)**, HALLMARK MYOGENESIS **(I)**, and HALLMARK PANCREAS BETA CELLS **(J)**.

To investigate the biological function differences associated with prognosis between high-risk and low-risk groups, we performed functional enrichment analysis. GSEA based on GO gene sets revealed that the high-risk group was significantly enriched in several cancer-related pathways, including HALLMARK E2F TARGETS, HALLMARK G2M CHECKPOINT, HALLMARK MTORC1 SIGNALING, HALLMARK MYC TARGETS V1, and HALLMARK MYC TARGETS V2 (**Figure 11A**). In contrast, the low-risk group exhibited significant enrichment in pathways associated with metabolism and detoxification, such as HALLMARK BILE ACID METABOLISM, HALLMARK XENOBIOTIC METABOLISM, HALLMARK COAGULATION, HALLMARK FATTY ACID METABOLISM, and HALLMARK ADIPOGENESIS (**Figure 11B**). Furthermore, through GSVA based on Hallmark gene sets, we observed that the high-risk group exhibited stronger activation in pathways related to HALLMARK MYC TARGETS V1, HALLMARK MYC TARGETS V2, and HALLMARK UNFOLDED PROTEIN RESPONSE. In contrast, the low-risk group showed more prominent activity in pathways such as HALLMARK COAGULATION, HALLMARK BILE ACID METABOLISM, and HALLMARK XENOBIOTIC METABOLISM (**Figure 11C**). Correlation analysis between riskscores and Hallmark pathway scores further corroborated these findings (**Figure 11D**), suggesting a strong association between risk scores and cancer-related biological processes and metabolic pathways. To assess whether these Hallmark pathway scores are linked to prognosis in HCC patients, we performed survival analysis. The results indicated that pathways such as HALLMARK DNA REPAIR, HALLMARK G2M CHECKPOINT, and HALLMARK PI3K AKT MTOR SIGNALING were positively correlated with poor prognosis (**Figures 11E–G**, **Supplementary Figures S4K–O**), while pathways like HALLMARK COAGULATION, HALLMARK MYOGENESIS, and HALLMARK PANCREAS BETA CELLS were associated with better prognosis (**Figures 11H–J**).

This analysis suggests that the biological functions of high-risk patients are primarily enriched in pathways related to cancer progression, such as tumor cell proliferation, DNA repair, and cell cycle regulation. Activation of these pathways likely drives tumor progression and contributes to poorer prognosis. In contrast, the biological functions of low-risk patients are mainly concentrated in metabolic pathways, including fatty acid and bile acid metabolism. The maintenance of these metabolic processes may help stabilize tumor growth and contribute to a better prognosis. Thus, significant differences in the active states of biological functions and related pathways between high-risk and low-risk patients may play a crucial role in driving the prognostic disparities observed between these two groups.

### 3.12 Mutation landscape and tumor heterogeneity in PMRS

**Figure 12:**
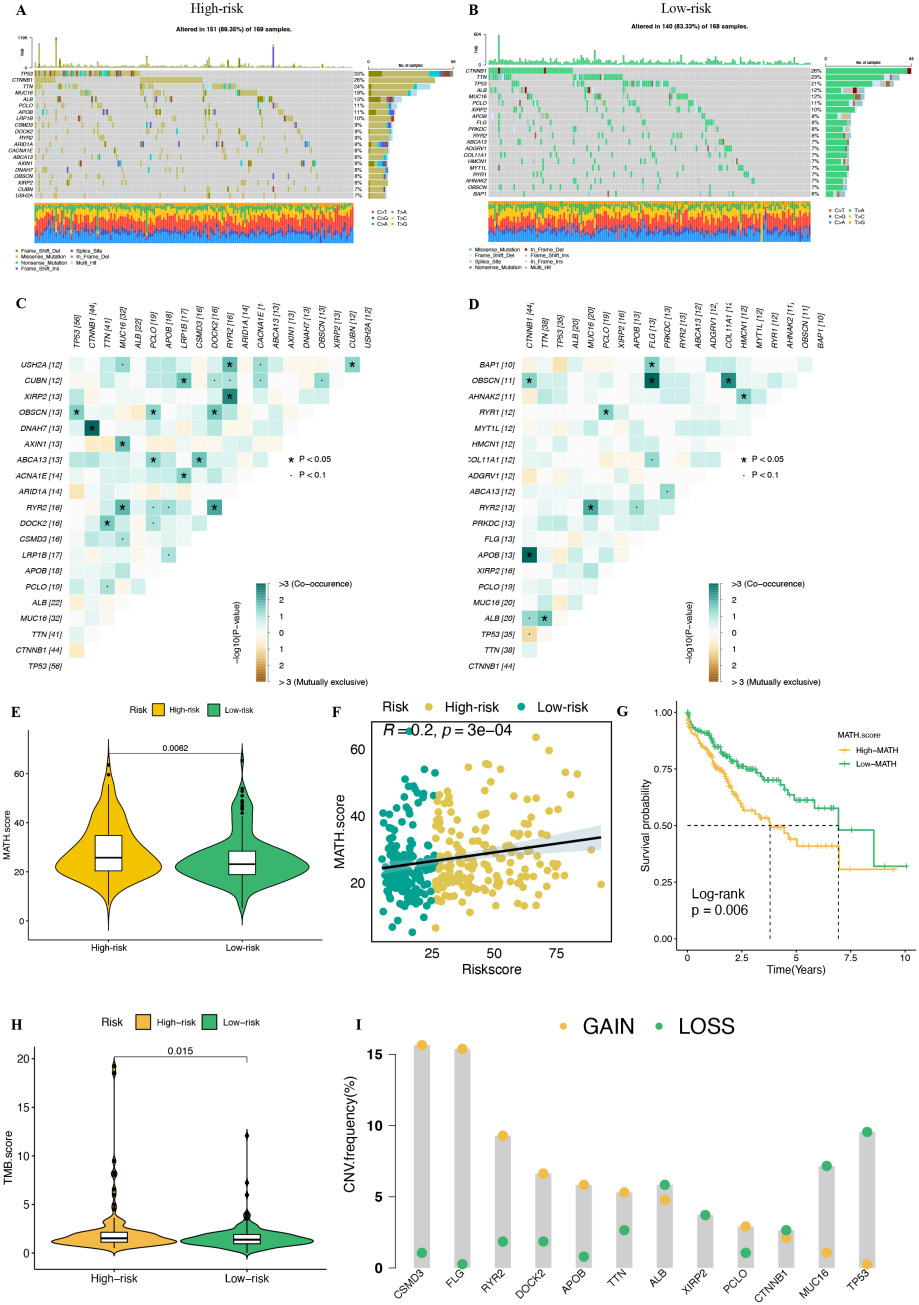
Distinct mutation landscapes between the high-risk and low-risk groups. **(A, B)** Waterfall plots of the top 20 genes by mutation frequency in the high-risk and low-risk groups. **(C, D)** Co-mutation and mutually exclusive mutation maps of the top 20 genes in the high and low risk groups. **(E)** The violin plot shows the difference in mutation allele tumor heterogeneity (MATH) scores between the high-risk and low-risk groups. **(F)** Spearman correlation analysis between MATH score and riskscore. **(G)** The Kaplan-Meier survival curve shows the OS differences between high and low MATH score groups. **(H)** Distribution of tumor mutational burden (TMB) in the high-risk and low-risk groups. **(I)** Distribution of CNV frequencies among DEGs between the high-risk and low-risk groups. * P <0.05.

To investigate the differences in genomic mutations between the high-risk and low-risk groups, we analyzed the mutation profiles of these two subgroups (**Figures 12A, B**, **Supplementary Figures S5A, B**). The results revealed significant differences in mutation spectra between the groups: TP53 mutations were predominant in the high-risk group, while CTNNB1 mutations were more prevalent in the low-risk group. Fisher’s exact test identified significant differences in the mutation frequencies of DOCK2, MAGI2, and PCDHA5 between the two groups (**Supplementary Figure S5C**, P < 0.01). Specifically, the high-risk group had a higher mutation frequency in DOCK2, while the low-risk group exhibited increased mutation frequencies in MAGI2 and PCDHA5. **Figures 12C, D** further illustrate that the high-risk group displayed a higher frequency of co-occurring mutations compared to the low-risk group.

ITH refers to the genetic differences present among various cellular populations within a tumor, typically resulting from the accumulation of mutations during tumor growth. ITH is closely associated with rapid tumor progression, metastasis, and resistance to chemotherapy, which can lead to treatment failure. To quantify ITH in HCC patients, we applied the MATH algorithm, with higher MATH scores indicating greater ITH. Our analysis revealed that the high-risk group had significantly higher MATH scores than the low-risk group (**Figure 12E**). Additionally, a significant positive correlation was observed between the MATH score and risk score (**Figure 12F**, R = 0.2, P < 0.05). Further investigation into the relationship between ITH and prognosis showed that patients with higher MATH scores had a poorer prognosis (**Figure 12G**). We also analyzed the tumor mutation burden (TMB) and found that the high-risk group had TMB values concentrated in the higher range, suggesting a greater accumulation of mutations in this group (**Figure 12H**).

Finally, we examined the copy number variations (CNV) of the top 20 genes with the most significant differences between the high-risk and low-risk groups. The results indicated that the high-risk group exhibited copy number gains in genes such as CSMD3, FLG, RYR2, DOCK2, APOB, and TTN, while showing copy number losses in genes like TP53, MUC16, CTNNB1, PCLO, and XIRP2 (**Figure 12I**). These CNV differences suggest that the high-risk group experiences greater genomic instability and tumor heterogeneity, which may be closely linked to tumor invasiveness, recurrence risk, and the regulation of the TIME.

### 3.13 Correlation between PMRS and TIME

**Figure 13:**
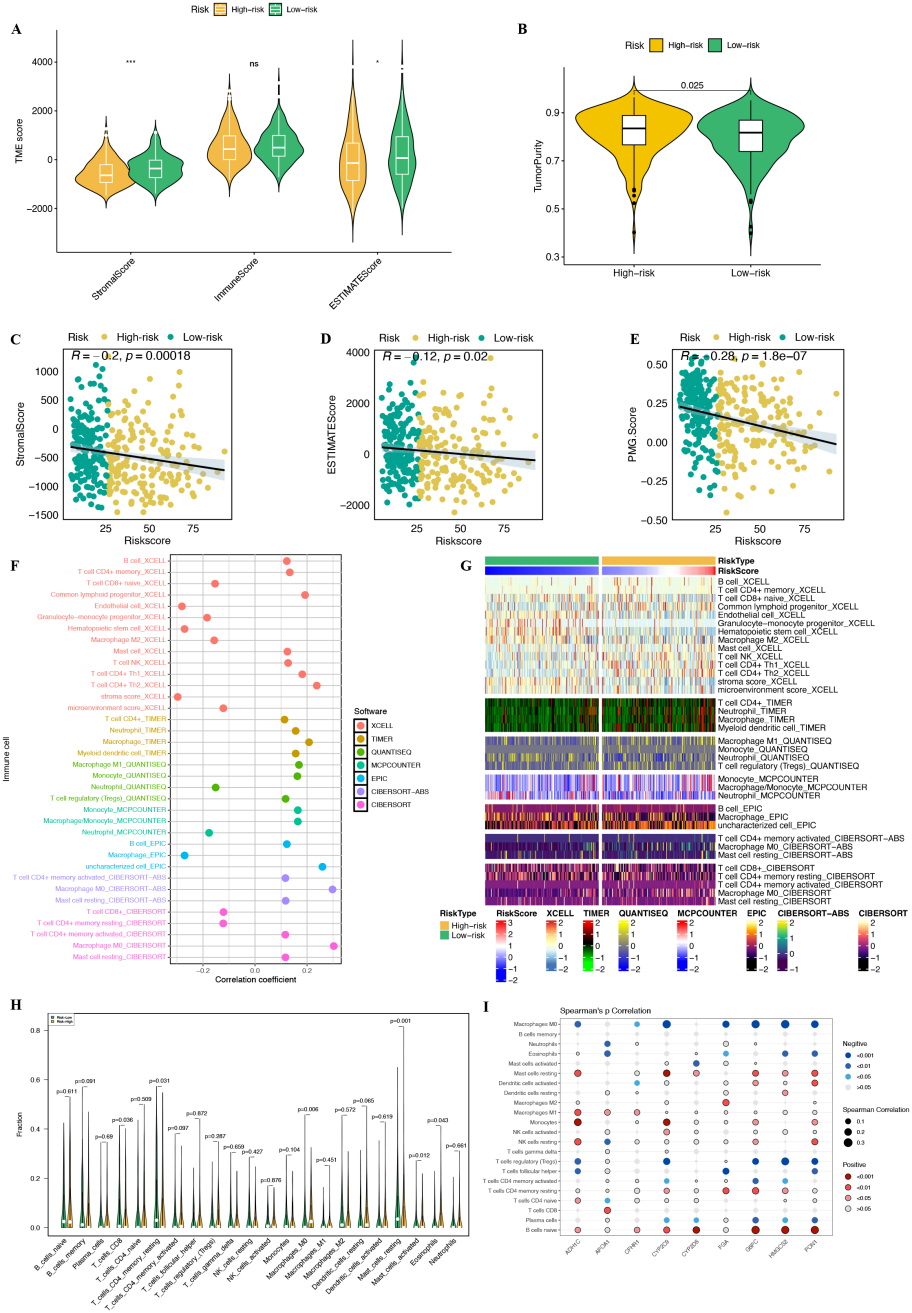
The immune landscape associated with PMRS in HCC. **(A, B)** The StromalScore, ImmuneScore, ESTIMATEScore, and TumorPurity were used to quantify the differences in immune status between the high-risk and low-risk groups. **(C-E)** Spearman correlation analysis between StromalScore, ESTIMATEScore, PMG score (PMGS), and riskscore. **(F, G)** Different immune cell infiltration patterns between the high-risk and low-risk groups. **(H)** Abundance of each TME-infiltrated cell type between the high-risk and low-risk groups, quantified using the CIBERSORT algorithm. **(I)** The association between TME-infiltrated cells and genes included in the PMRS. NS, not statistically significant; *p < 0.05; **p < 0.01; ***p < 0.001.

To assess the relationship between different risk subgroups of HCC patients and immune cell infiltration, we employed the ESTIMATE algorithm to calculate stromal scores, immune scores, ESTIMATE scores, and tumor purity across the different risk subgroups. The results indicated that the high-risk group had significantly lower stromal and estamate scores (**Figure 13A**), while exhibiting higher tumor purity (**Figure 13B**). Correlation analysis revealed significant negative associations between the riskscore and both stromal score, estamate score, and the PMGS (**Figures 13C–E**).

To further explore immune cell infiltration, we used several immune cell quantification algorithms, including XCELL, TIMER, QUANTISEQ, MCPCOUNTER, EPIC, CIBERSORT-ABS, and CIBERSORT, to assess the abundance of immune cell populations in each sample. The correlation analysis results between the risk score and various immune cell types are shown in **Figure 13F**, while a heatmap illustrating the expression levels of different immune cell types in high-risk and low-risk groups is provided in **Figure 13G**. To examine specific differences in immune cell infiltration between the risk subgroups, we applied the CIBERSORTS algorithm, which allowed us to compare immune cell abundances across the risk groups (**Figure 13H**, **Supplementary Table S15**). This analysis revealed that the high-risk group was enriched for Macrophages M0 and Eosinophils, while the low-risk group showed higher infiltration of T cells CD8, T cells CD4 memory resting, Mast cells resting and Mast cells activated. Further investigation into the relationship between immune cell types and OS in HCC patients indicated that the infiltration of specific immune cell types was closely associated with prognosis and disease progression. Notably, T cells CD8, Macrophages M0, T cells CD4 memory resting, and Eosinophils were significantly correlated with HCC prognosis (**Supplementary Figures S6A–K**).

Lastly, we explored the correlation between the nine genes in the PMRS model and the abundance of tumor-infiltrating immune cells. Our analysis revealed that APOA1 was highly positively correlated with the abundance of CD8+ T cells, while ADH1C, CYP2C9, FGA, G6PC, HMGCS2, and PON1 showed strong negative correlations with the abundance of macrophages M0 (**Figure 13I**).

### 3.14 Relationship between PMRS, immune-related pathways, and immunotherapy response

**Figure 14:**
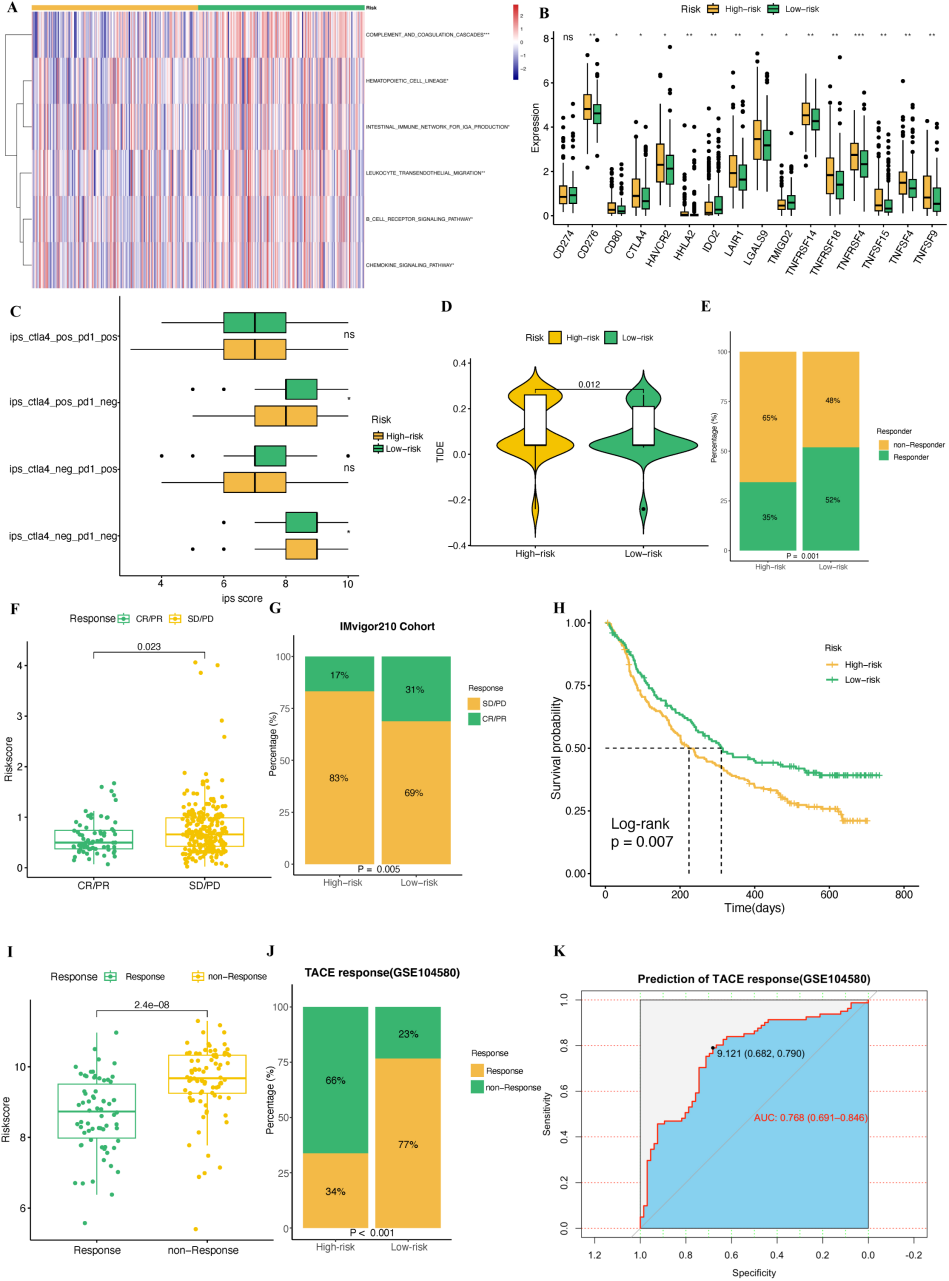
Immunotherapy sensitivity analysis between the high-risk and low-risk groups. **(A)** Immune-related pathways’ activity showing a significant difference between high- and low-risk groups. **(B)** The expression of immune checkpoints in high- and low-risk groups. **(C)** IPS score comparison between the high-risk and low-risk groups. **(D)** Comparison of TIDE scores between the high-risk and low-risk groups. **(E)** Comparison of non-Responders and Responders to immunotherapy based on the TIDE analysis between the high and low risk groups. **(F)** A boxplot depicting the difference in riskscore between patients with CR/PR and those with SD/PD in the IMvigor210 cohort. **(G)** The proportion of CR/PR or SD/PD patients, who received immunotherapy, in high- and low-risk groups of the IMvigor210 cohort. **(H)** The Kaplan-Meier survival curve shows the difference in OS between high-risk and low-risk groups in the IMvigor210 cohort. **(I)** A boxplot depicting the difference in risk scores between patients with TACE response and those with TACE non-response in the GSE104580 cohort. **(J)** The proportion of TACE response and TACE non-response patients in the high-risk and low-risk groups of the GSE104580 cohort. **(K)** ROC curve to predict TACE treatment response using the riskscore.

To explore the immune characteristics of different risk subgroups, we applied the ssGSEA algorithm to calculate activity scores for immune-related pathways. The results indicated that the high-risk group exhibited lower activity in several immune-related pathways, including COMPLEMENT AND COAGULATION CASCADES, HEMATOPOIETIC CELL LINEAGE, IINTESTINAL IMMUNE NETWORK FOR IGA PRODUCTION, LEUKOCYTE TRANSENDOTHELIAL MIGRATION, B CELL RECEPTOR SIGNALING PATHWAY, and CHEMOKINE SIGNALING PATHWAY (**Figure 14A**). Previous studies have shown that high expression of immune checkpoints can enhance tumor cell sensitivity to immune checkpoint inhibitors (ICIs) treatment (30–32). Consistent with these findings, we observed that most immune checkpoints, including CTLA4, were more highly expressed in the high-risk group, with the exceptions of CD274, IDO2, and TMIGD2 (**Figure 14B**).

To further validate these results, we examined immune response scores (IPS) from the TCIA database. The analysis revealed that the high-risk group had significantly higher IPS scores in both the CTLA4+/PD1- and CTLA4-/PD1- groups, suggesting that these patients may benefit more from CTLA4-targeted treatments (**Figure 14C**). Additionally, TIDE score analysis indicated that the high-risk group exhibited higher TIDE scores and a greater proportion of non-responder patients, which is indicative of immune escape (**Figures 14D, E**). These results suggest that while high-risk patients show elevated immune activity, they are more prone to immune escape mechanisms.

To further evaluate the potential of riskscore in predicting immunotherapy response, we analyzed data from the IMvigor210 cohort, which was treated with Atezolizumab. Basing the PMRS, we classified patients into high-risk and low-risk groups based on their riskscore. The high-risk group showed a higher proportion of patients with SD/PD (**Figures 14F, G**). Survival analysis confirmed that high-risk patients had worse prognosis compared to the low-risk group (**Figure 14H**). Finally, we validated the predictive power of the riskscore in the GSE104580 cohort. The results demonstrated that the high-risk group had a significantly higher proportion of non-responder patients (**Figures 14I–J**). Furthermore, in predicting the response to TACE treatment, the riskscore showed an AUC of 0.768 (CI: 0.691-0.846), confirming that the riskscore can be a reliable predictive tool for TACE treatment outcomes in HCC patients (**Figure 14K**).

### 3.15 Drug sensitivity analysis

**Figure 15:**
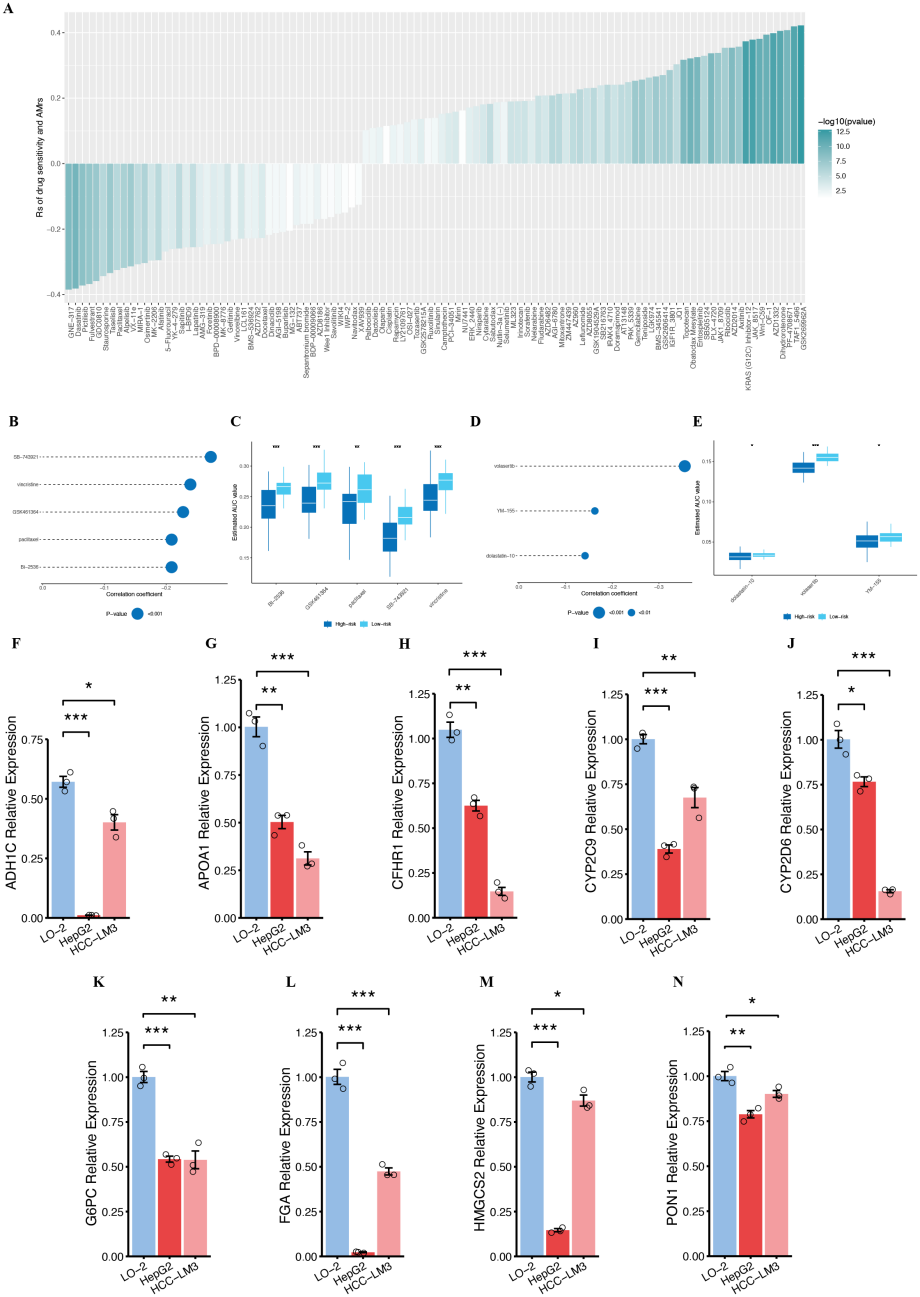
Association between the PMRS and drug sensitivity and validation of the genes. **(A)** Analyzing the association between IC50 values and the riskscores in patients with HCC. **(B-E)** Analysis of correlation and differences in sensitivity to drugs among potential medications derived from the CTRP and PRISM datasets. **(F-N)** Validation of the expression of ADH1C **(F)**, APOA1 **(G)**, CFHR1 **(H)**, CYP2C9 **(I)**, CYP2D6 **(J)**, G6PC **(K)**, FGA **(L)**, HMGCS2 **(M)**, PON1 **(N)**, NS, not statistically significant; *P < 0.05; **P < 0.01; ***P < 0.001.

In this drug sensitivity analysis, we aimed to optimize therapeutic strategies for HCC patients by focusing on treatment targets and drugs that were significantly associated with risk scores. We analyzed the IC50 values of 198 compounds from the GDSC database and performed Spearman correlation and differential analysis between high-risk and low-risk groups. The filtering criteria were set to |cor| > 0.1 and P < 0.05. Our findings revealed that the IC50 values of 5-Fluorouracil, Afatinib, and Gefitinib were negatively correlated with the risk score, suggesting that high-risk patients may benefit more from these treatments. In contrast, Sorafenib, Gemcitabine, and Axitinib showed positive correlations with the risk score, indicating that low-risk patients are more sensitive to these drugs (**Figure 15A**).

To identify additional candidate drugs with higher sensitivity in high-risk patients, we used drug response data from the CTRP and PRISM databases. First, we conducted a differential drug response analysis between the high-risk group (top decile) and low-risk group (bottom decile), with a log2FC > 0.10. Subsequently, we performed Spearman correlation analysis between the AUC values and the riskscore, identifying compounds that were significantly negatively correlated with the riskscore (**Figures 15B–E**). Based on these analyses, we identified five compounds from the CTRP database (SB-743921, Vincristine, GSK461364, Paclitaxel, and BI-2536) and three compounds from the PRISM database (Volasertib, YM-155, and Dolastatin-10) that exhibited a consistent negative correlation with riskscore. These compounds showed lower AUC values in the high-risk group, suggesting their potential efficacy in treating HCC patients with higher riskscore.

### 3.16 Relative expression of PMRS model genes’ RNA

To explore the expression profiles of the genes included in the PMRS model, we compared the expression levels of the nine genes in normal and tumor tissues using the UALCAN online tool (https://ualcan.path.uab.edu/index.html). The analysis revealed that the average expression of these genes was significantly higher in normal than in primary tumor (**Supplementary Figures S7A–I**). Additionally, survival analysis indicated that higher expression levels of these genes were associated with better prognosis in HCC patients (**Supplementary Figures S7J–R**). Furthermore, we evaluated the expression of these nine genes in three cell lines: one normal hepatocyte cell line (LO-2) and two HCC cell lines (HepG2 and HCC-LM3). The results showed that the expression of ADH1C, APOA1, CFHR1, CYP2C9, CYP2D6, G6PC, FGA, HMGCS2, and PON1 was significantly downregulated in the tumor cell lines compared to the normal LO-2 cells (**Figures 15F–N**).

## 4 Discussion

HCC is often diagnosed at advanced stages due to the absence of specific clinical symptoms, leading to delayed detection. As a result, many patients miss the opportunity for curative surgical treatments at diagnosis. Despite substantial advances in cancer prevention, early detection, and treatment strategies over the past few years, the prognosis for HCC patients remains poor, characterized by a low survival rate (33). This underscores the critical need for the identification of novel therapeutic targets to improve treatment outcomes for HCC patients.

Polyamines are essential tumor metabolites that contribute to immune suppression and are closely linked to tumor growth and progression (34). During the early stages of tumorigenesis, various oncogenic pathways increase the demand for polyamines, leading to metabolic dysregulation (35). Elevated polyamine levels are therefore considered crucial for tumor transformation and progression. Polyamines and their metabolites have also been identified as potential biomarkers in various cancers. For example, Xu et al. observed significant alterations in polyamine metabolites in the plasma and urine of lung and liver cancer patients, with notable differences in polyamine concentrations (36). Similarly, Giskeødegård et al. identified specific metabolites in prostate cancer as potential invasive biomarkers (37). Asai et al. discovered that salivary metabolites, including polyamines, could serve as valuable screening tools for pancreatic cancer (38). Furthermore, Nakajima et al. demonstrated that combining polyamine profiles with machine learning techniques could enhance colorectal cancer screening (39). However, the role of PMRG in HCC remains inadequately explored. This study represents the first comprehensive investigation into the potential applications of PMRG in HCC using a multi-omics approach, offering new insights for prognosis prediction and personalized medicine in HCC.

Machine learning (ML) techniques, particularly multimodal machine learning, present exciting opportunities for integrating multi-source data and constructing more accurate predictive models, owing to their advanced data processing and pattern recognition capabilities (40). As a significant branch of artificial intelligence, ML has shown remarkable potential in the diagnosis, prognosis prediction, and treatment of liver cancer (41). One commonly used regression method in high-dimensional data analysis is the least absolute shrinkage and selection operator (Lasso), which is particularly effective for feature selection and managing model sparsity. In cancer research, combining Lasso with random survival forests (RSF) has become a powerful approach for constructing prognostic models and predicting postoperative recurrence risk. These integrated models not only improve prognosis prediction accuracy but also offer valuable insights into personalized treatment and follow-up strategies (42–45). In this study, we utilized the Lasso+RSF algorithm to demonstrate its strong predictive capability for the prognosis of HCC patients. Through comprehensive internal and external validation analyses, we confirmed the robustness of the riskscore derived from the PMRS in predicting the prognosis of HCC patients.

In this study, we constructed a 9-gene polyamine metabolism-related signature (PMRS) based on 101 machine learning algorithms, utilizing genes associated with polyamine metabolism to predict the prognosis of HCC patients. These genes are involved in various metabolic pathways and tumorigenesis processes, showing promise as prognostic biomarkers. Alcohol dehydrogenase 1C (ADH1C), a member of the alcohol dehydrogenase family, plays a role in the metabolism of ethanol, retinol, fatty alcohols, hydroxysteroids, and lipid peroxidation products. Studies have shown that ADH1C expression is significantly downregulated in HCC cells, with its silencing promoting cell proliferation and migration (46). Low ADH1C expression is linked to the activation of tumorigenic pathways, while higher expression is associated with better prognosis in HCC (47–49). Apolipoprotein A1 (ApoA1), the main protein component of high-density lipoprotein (HDL), has anti-inflammatory, immune-regulatory, and antioxidant properties. Its expression decreases during HCC progression and is significantly correlated with better prognosis in HCC patients (50). Complement factor H-related 1 (CFHR1) is a secreted protein in the complement factor H family. Homozygous deletion of CFHR1 has been associated with acute myeloid leukemia, suggesting its role in immune regulation (51). Cytochrome P450 2D6 (CYP2D6) is involved in drug metabolism, cholesterol, and steroid synthesis. Studies indicate that heterozygous deletion of CYP2D6 increases HCC sensitivity to tazobactam, highlighting its potential as a therapeutic target (52). Fibrinogen alpha chain (FGA) is a glycoprotein involved in blood coagulation. Research by Han et al. showed that FGA has anti-metastatic effects by inhibiting epithelial-mesenchymal transition (EMT) and HCC cell migration through the PI3K/AKT pathway, reducing metastasis (53). Glucose-6-phosphatase (G6PC) is crucial for gluconeogenesis and glycogenolysis, and its downregulation in HCC tissues is linked to tumor development and metastasis. G6PC expression impacts glucose metabolism and homeostasis, playing a key role in HCC progression (54). 3-Hydroxymethylpentanediol-CoA synthase 2 (HMGCS2) is the rate-limiting enzyme for ketone production, which synthesizes ketone bodies, β-hydroxybutyrate (β-HB) and acetic acid. Suk et al. found that HMGCS2 expression can affect the sensitivity of liver cancer cells to sorafenib (55). The enzyme encoded by the para-hydroxyl oxygenase 1 (PON1) gene is an aryl esterase, and PON1 is involved in various oxidative stress-related diseases (56). Despite this, the regulatory mechanism of this gene in liver cancer remains unclear and deserves further exploration and research in the future.

Omics approaches have become essential tools in cancer research, particularly for identifying and characterizing diagnostic and prognostic biomarkers. By integrating multi-dimensional data from genomics, transcriptomics, proteomics, and metabolomics, researchers can gain a more comprehensive understanding of the mechanisms underlying cancer initiation and progression. This approach provides valuable scientific evidence for early diagnosis, precision treatment, and prognostic evaluation (57, 58). The discovery and identification of various biomarkers have significantly advanced cancer diagnosis and prognosis. For example, biomarkers such as AFP, HER2, PSA, and EGFR are widely used in liver cancer, breast cancer, prostate cancer, and non-small cell lung cancer (NSCLC), respectively (58–61). Omics technologies enhance our understanding of the molecular mechanisms driving diseases. By performing risk stratification and molecular subtype analysis based on omics data, clinical treatment decisions can be better guided, facilitating personalized treatment strategies (62). For instance, multi-omics analysis of triple-negative breast cancer (TNBC) has revealed three distinct metabolic subtypes: lipid synthesis, glycolysis, and mixed types, each with unique metabolic characteristics. The lipid synthesis subtype is more responsive to fatty acid synthase inhibitors, while the glycolysis subtype shows greater sensitivity to glycolytic inhibitors (63). In esophageal cancer, saliva-based sequencing studies developed exosomal small RNA signatures that serve as preoperative biomarkers for diagnosis and prognostic risk stratification, helping to identify patients who would benefit from adjuvant therapy (64). Similarly, NSCLC has been stratified into different subtypes based on metabolic features, with varying sensitivity to metabolism-related drugs (65). In gastric cancer, a machine learning-based diagnostic model using serum exosomal ncRNAs has been developed, allowing for early-stage detection and revealing the key role of DGCR9 in gastric cancer progression, along with its potential as a therapeutic target (66). Furthermore, metabolic reprogramming signatures in colorectal cancer research have been used to identify new therapeutic targets, driving the development of targeted therapies based on these signatures (67). However, the application of multi-omics analysis in the molecular characterization of HCC remains underexplored. In our study, we leveraged multi-omics approaches to establish a novel risk signature for predicting the prognosis of HCC patients and conducted risk stratification, thereby facilitating early prediction, targeted prevention, and personalized treatment strategies. Furthermore, our goal is to apply multi-omics methodologies to uncover the molecular mechanisms underlying these signatures, providing a molecular foundation for understanding the relationship between PMRG and prognosis, as well as immune treatment response in HCC.

Increasing evidence suggests that tumor cells promote the formation of an immunosuppressive microenvironment by enhancing polyamine synthesis and metabolism (68). These immunosuppressive effects help cancer cells evade immune surveillance, thereby facilitating tumor progression. For instance, TP53 inhibits the urea cycle, leading to ammonia accumulation, which directly downregulates the translation of ODC1 mRNA. This results in reduced ODC activity, impairing polyamine synthesis and slowing tumor cell proliferation. Thus, polyamine metabolism in HCC can serve as a valuable marker for assessing tumor malignancy (69). Our study found that the clust1 has a poorer prognosis compared to the clust2. Although clust1 exhibits higher immune activity than clust2, TIDE analysis reveals that clust1 has stronger immune evasion capabilities. This suggests that the clust1 is more likely to foster an immunosuppressive microenvironment. Furthermore, the PMRS model results show that the high-risk group not only has lower immune activity but also higher TIDE scores and tumor purity, further supporting the potential role of the PMRS in immune evasion and treatment response.

Casero et al. discovered that polyamines possess anti-inflammatory and immunosuppressive properties, suggesting that modulating polyamine levels could potentially enhance the immune response in tumors (70). Conversely, Holbert et al. highlighted that alterations in polyamine levels may be associated with the development of “immune desert tumors,” which generally show poor responses to immune checkpoint inhibitors (71). In our study, we found that the clust1 exhibited higher expression levels of immune checkpoints. Moreover, the IPS score for the ips_ctla4_pos_pd1_neg subgroup was significantly elevated. These findings suggest that clust1 may play a pivotal role in immune regulation and could influence tumor responsiveness to immunotherapy. Future research should further investigate the relationship between polyamine metabolism and immune evasion mechanisms, as well as explore its potential therapeutic implications for tumor immunotherapy.

In the PMRS model, the high-risk group is closely associated with carcinogenic pathways, while the low-risk group is predominantly linked to metabolic pathways. The risk score is significantly correlated with the enrichment of various immune-related pathways. Notably, patients with higher risk scores exhibit elevated expression levels of immune checkpoints and higher IPS scores for ips_ctla4_pos_pd1_neg, further suggesting potential differences in immune treatment responses between the two risk groups. High-risk patients undergoing anti-PD-L1 therapy are more likely to experience SD or progression PD, whereas low-risk patients tend to achieve CR or PR. Currently, transcatheter arterial chemoembolization (TACE) is considered the standard first-line treatment for intermediate-stage HCC, with an objective response rate (ORR) of 52.5% according to recent studies (72). Thus, developing reliable biomarkers to predict the efficacy of TACE treatment is essential. Our study demonstrates that the PMRS has strong predictive power in assessing TACE efficacy, highlighting its potential as a tool for guiding clinical decision-making in HCC treatment.

Given the significant role of polyamines in shaping the tumor immune microenvironment and their broad impact on both tumor and immune cells, therapies aimed at regulating polyamine levels hold promise as a novel strategy in cancer treatment. Polyamine blockade therapy (PBT) has emerged as a potential adjunctive approach to enhance the efficacy of chemotherapy and immunotherapy across various cancer types (70, 73, 74). However, despite the advancements in polyamine-targeted therapies in other cancers, their application in HCC remains relatively underexplored.

In this study, we integrated multi-omics data and performed comprehensive bioinformatics analysis to identify key genes associated with polyamine metabolism that could serve as potential therapeutic targets in HCC. These targets provide a promising foundation for the development of novel, precise, and effective targeted therapies.

## 5 Limitations of the study

Despite the promising findings from our study, several limitations warrant consideration. First, although we have evaluated and validated the PMRS features across the training set, testing set, and external validation set, larger-scale, multi-center prospective studies are essential to further confirm and generalize our results. Second, additional *in vitro* and *in vivo* studies are needed to more thoroughly investigate the biological functions of polyamine metabolism-related genes in HCC, as these studies will provide a deeper understanding of their role in tumorigenesis and treatment responses.

## 6 Conclusion

In this study, we utilized consensus clustering based on the expression of PMRG to stratify the cohort into two distinct clusters. We identified significant differences in immune features, molecular characteristics, and the TME between these clusters, underscoring their potential utility in the stratification of HCC patients. Additionally, we developed the PMRS model, which demonstrates robust predictive capability for the prognosis and immune treatment response of HCC patients across multiple datasets. The model also proved effective in predicting the efficacy of TACE. Our findings lay a strong theoretical foundation for the development of personalized treatment strategies tailored to the unique molecular and immune profiles of HCC patients.

## Data Availability

The datasets presented in this study can be found in online repositories. The names of the repository/repositories and accession number(s) can be found in the article/**Supplementary Material**.
