# Supplementary material for: Multi-omics identification of a polyamine metabolism related signature for hepatocellular carcinoma and revealing tumor microenvironment characteristics
Source: Front Immunol. 2025 Apr 22;16:1570378. doi: 10.3389/fimmu.2025.1570378 (PMC12052762; doi:10.3389/fimmu.2025.1570378)
Supplement: Supplementary file 1 [file DataSheet1.pdf]

Multi-omics identification of a polyamine metabolism related signature for hepatocellular carcinoma and revealing tumor microenvironment characteristics

Yuexi Yu[1]*, Huiru Liu[1]*, Kaipeng Liu[2]*, Meiqi Zhao[3], Yiyan Zhang[1], Runci Jiang[1], Fengmei Wang[1#]

*These authors share first authorship.

[#] This author is the corresponding author.

[1] Department of gastroenterology &hepatology, Tianjin First Center Hospital, Tianjin Key Laboratory for Organ Transplantation, Tianjin Key Laboratory of Molecular Diagnosis and Treatment of Liver Cancer, Tianjin Medical University, Tianjin 300192, PR China.

[2] Department of Hepatobiliary Oncology, Liver Cancer Center, Tianjin Medical University Cancer Institute & Hospital, National Clinical Research Center for Cancer, Key Laboratory of Cancer Prevention and Therapy, Tianjin's Clinical Research Center for Cancer, Tianjin Medical University, Tianjin, China.

[3] Department of Hepatobiliary Oncology, Liver Cancer Center, Tianjin Medical University Cancer Institute & Hospital, National Clinical Research Center for Cancer, Key Laboratory of Cancer Prevention and Therapy, Tianjin's Clinical Research Center for Cancer, Nankai University, Tianjin, China.

Contact information

Fengmei Wang, Department of gastroenterology &hepatology, Tianjin First Center Hospital, Tianjin Key Laboratory for Organ Transplantation, Tianjin Key Laboratory of Molecular Diagnosis and Treatment of Liver Cancer, Tianjin Medical University, Tianjin 300192, PR China. Email: -wangfengmeitj@126.com

Supplementary Figures:

Figure S1. Analysis of the tumor immune microenvironment between clust1 and clust2 subtypes.

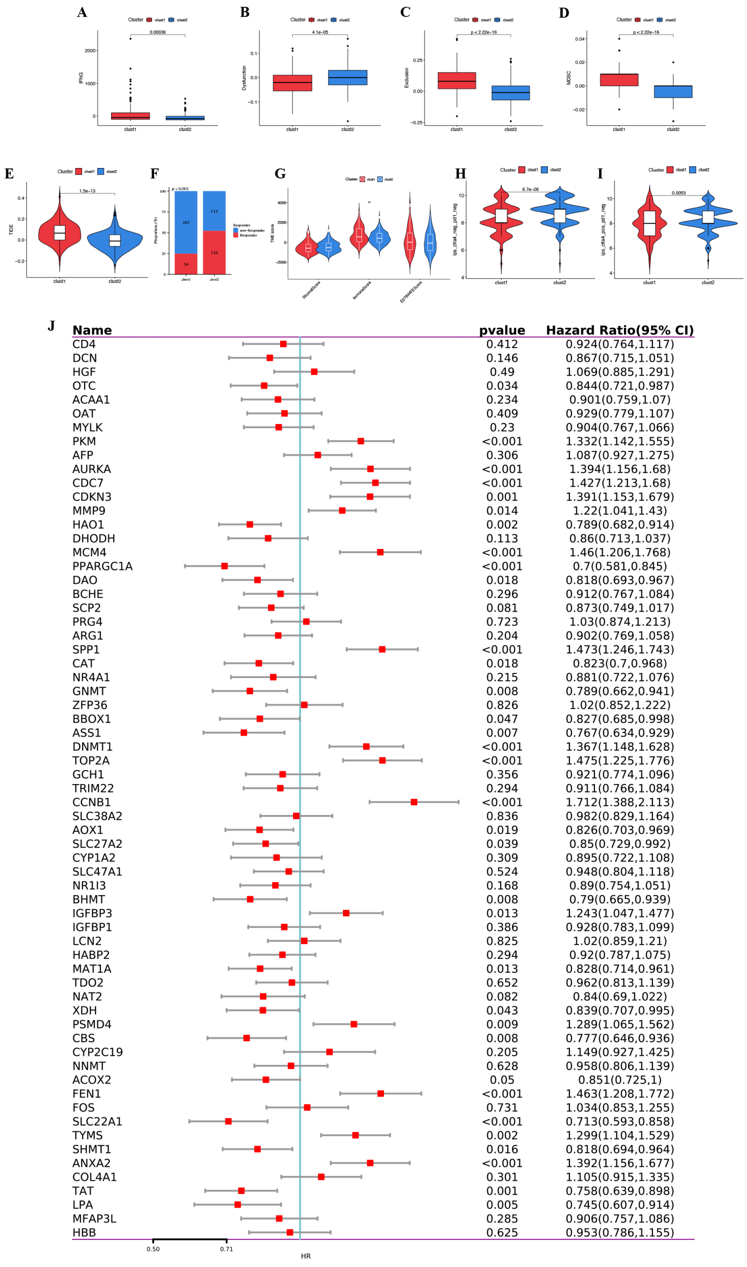

(A-F) Differences in IFNG, dysfunction, exclusion, MDSC, and TIDE between clust1 and clust2 subtypes.

(G) A violin plot comparing StromalScore, ImmuneScore, and ESTIMATEScore between the clust1 and clust2 subtypes.

(H-I) IPS score comparison between the clust1 and clust2 subtypes.

(J) The forest plot shows the univariate Cox regression analysis of PMRG among 65 genes.

Figure S2. Identification of the PMRG in single-cell and bulk transcriptomes.

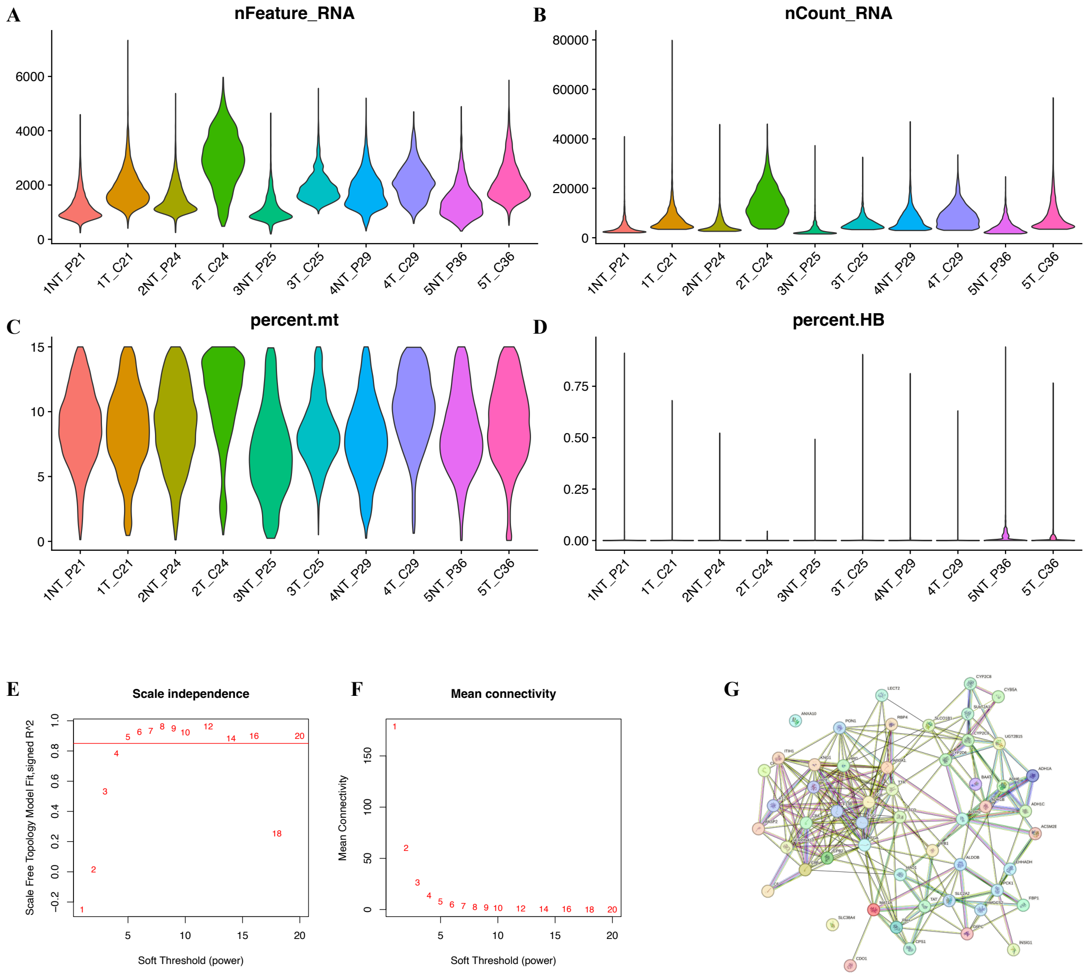

(A-D) nFeature_RNA, nCount_RNA, percent.mt, and percent.HB in samples after filtration.

(E-F) The determination of the optimal soft threshold in WGCNA analysis.

(G) Protein-protein interaction network of the 53 PMRG.

Figure S3. Riskscores are associated with poor progression in HCC within the TCGA cohort.

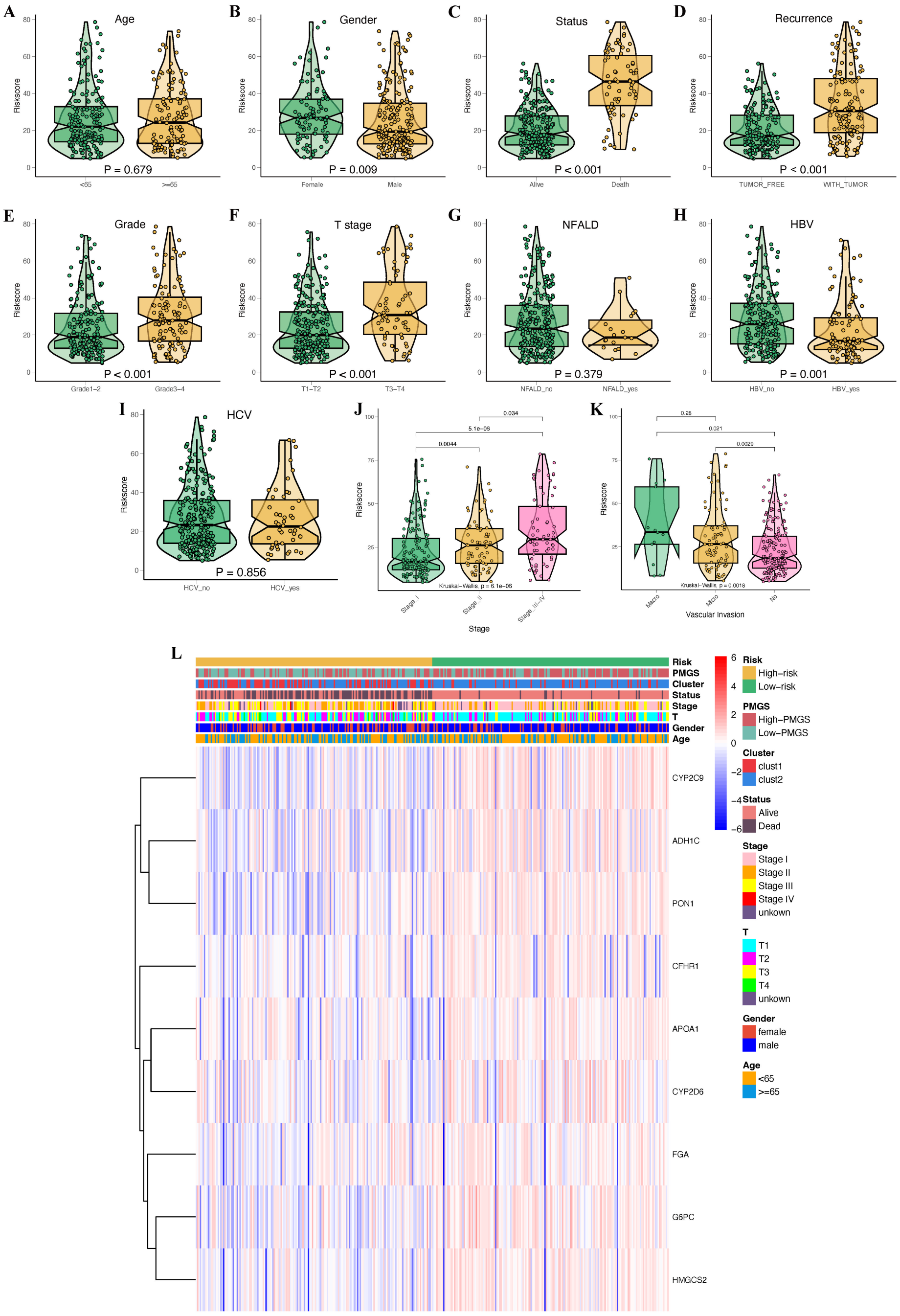

(A-K) Riskscores are not associated with Age (A), NFALD (G), or HCV (I), but are closely related to Gender (B), Status (C), Recurrence (D), Grade (E), T stage (F), HBV(H), Stage (J), and Vascular Invasion (K).

(L) Clinical feature heatmap.

Figure S4. Riskscores are associated with poor progression in HCC within the GSE14520 cohort.

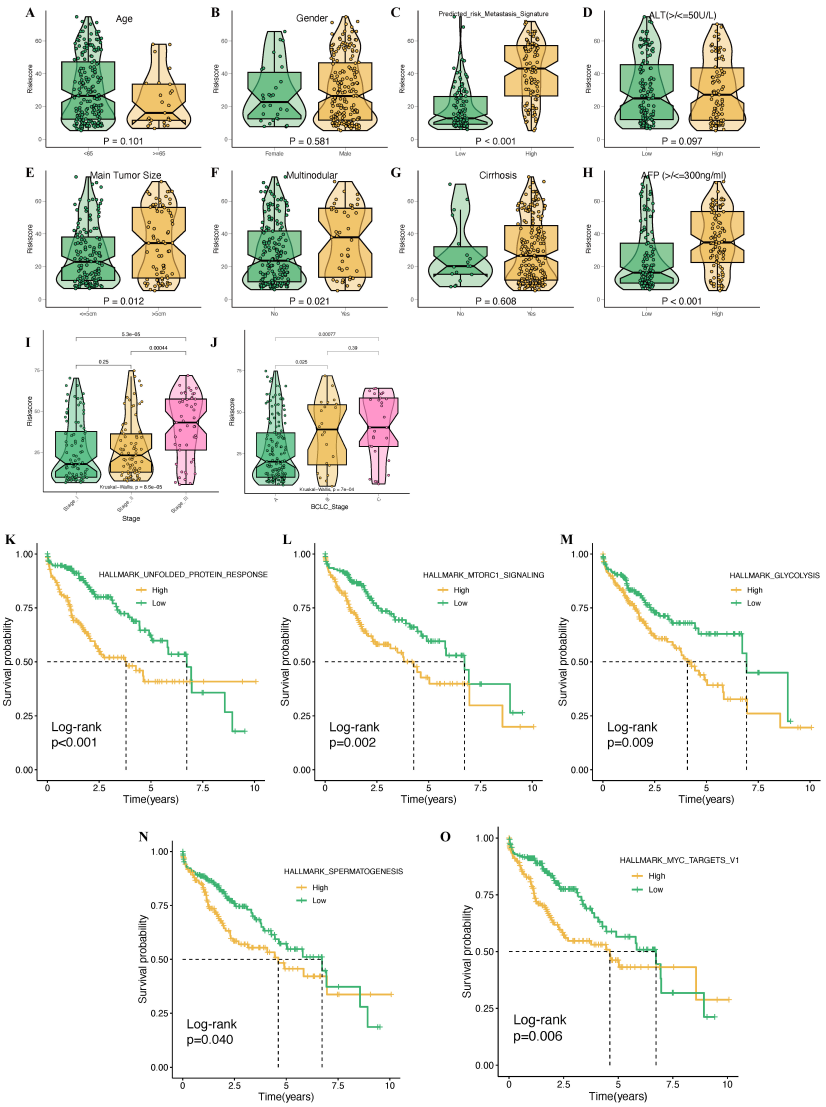

(A-K) Riskscores are not associated with Age (A), Gender (B), ALT(>/<=50U/L) (D), or Cirrhosis(G), but are closely related to Predicted_risk_Metastasis_Signature (C), Main Tumor Size (E), Multinodular (F), AFP (>/<=300ng/ml) (H), Stage (I), and BCLC_Stage (J).

(K-O) Kaplan–Meier survival plots showing significant correlations between OS and GSVA scores for HALLMARK UNFOLDED PROTEIN RESPONSE(K), HALLMARK MTORC1 SIGNALING(L), HALLMARK GLYCOLYSIS(M), HALLMARK SPERMATOGENESIS(N) and HALLMARK MYC TARGETS V1(O)

Figure S5. Mutation characteristics in high-risk and low-risk groups.

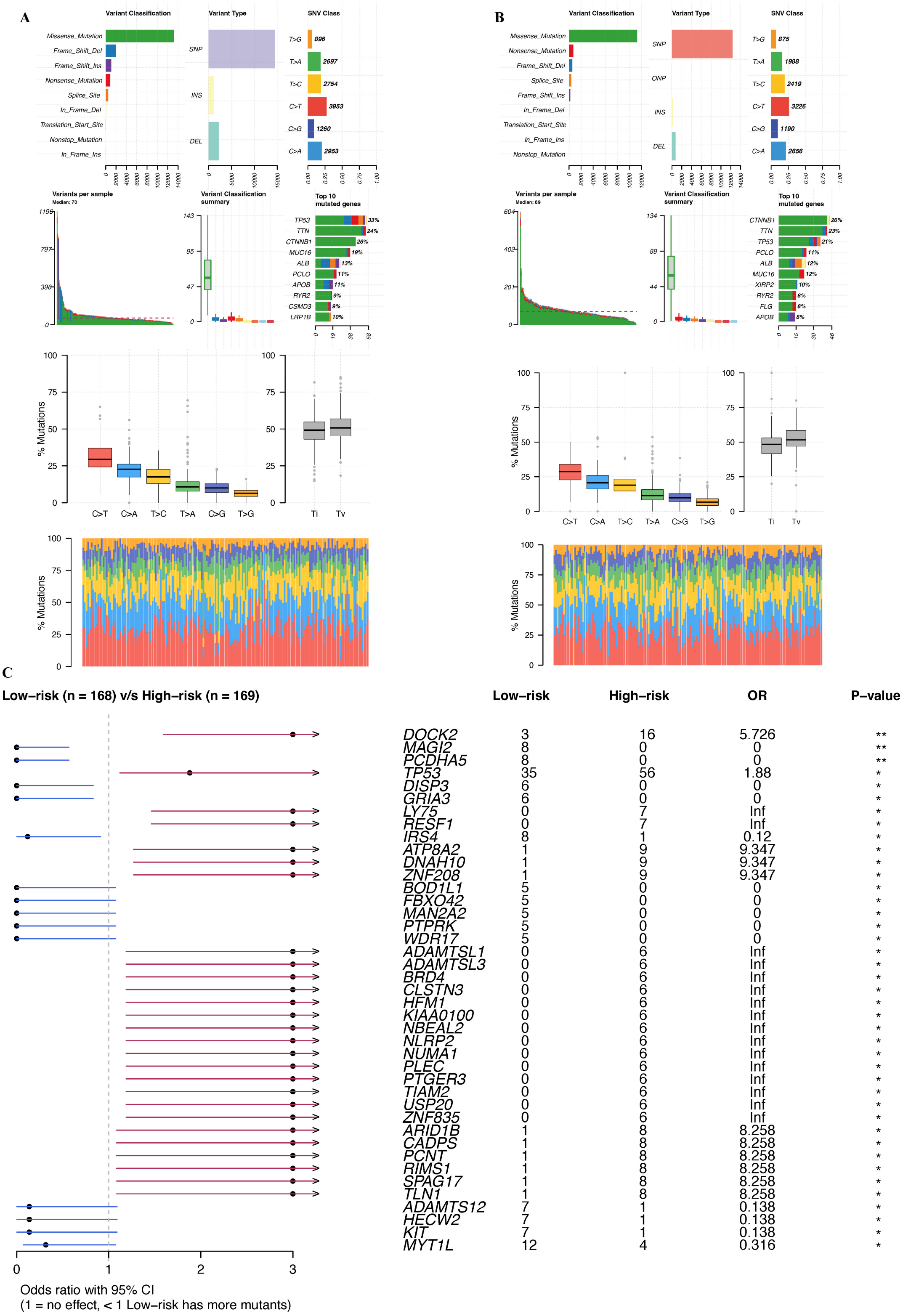

(A-B) MAF-summary plots of the somatic mutation showing the difference between the high-risk group (A) and low-risk group (B).

(C) The difference in gene mutation frequency between high- and low-risk groups.

Figure S6.

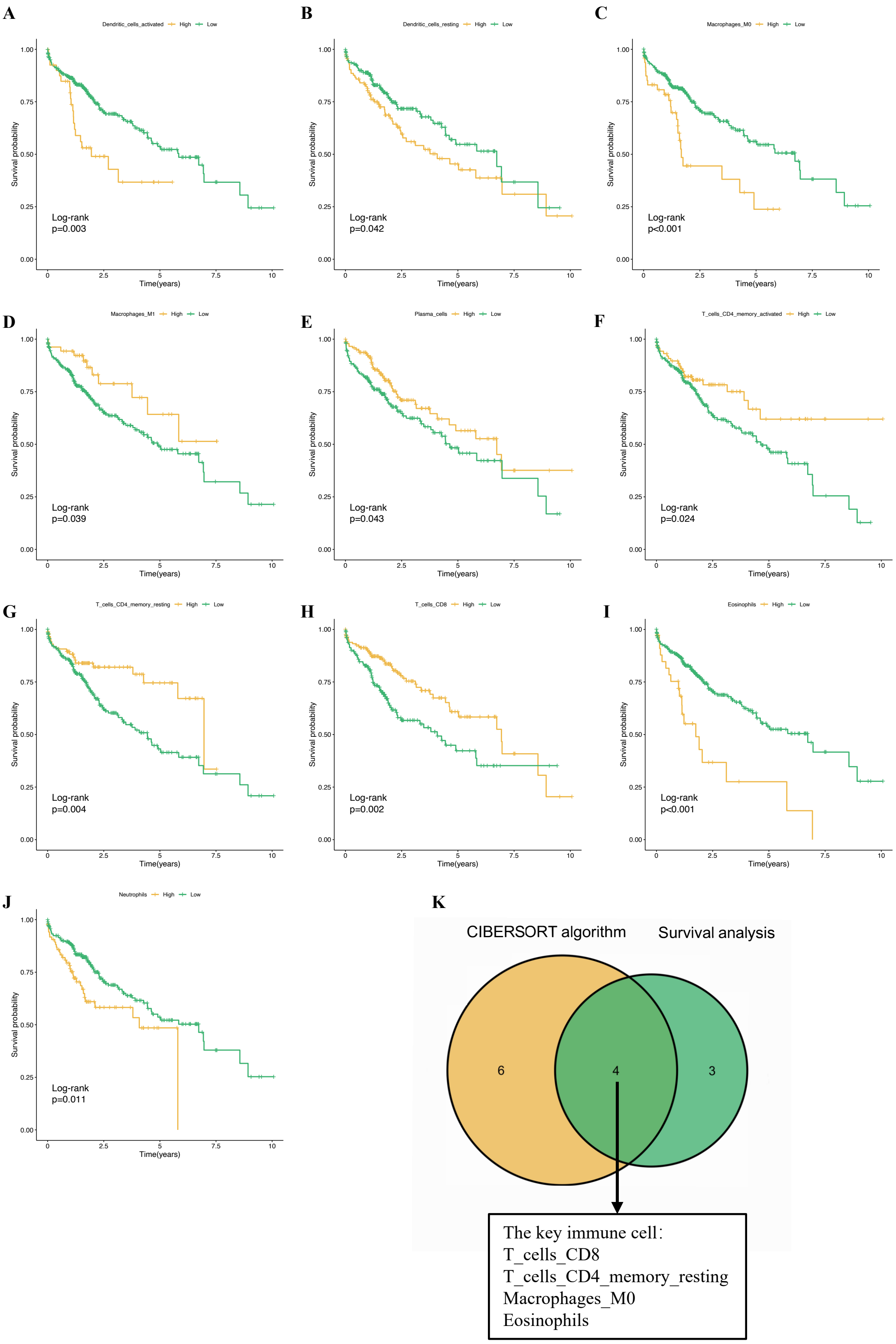

(A-J) Kaplan–Meier survival analysis based on the abundance of different immune cells.

(K) Venn plot showing the intersecting TME-infiltrated cell types of differential analysis and survival analysis.

Figure S7.

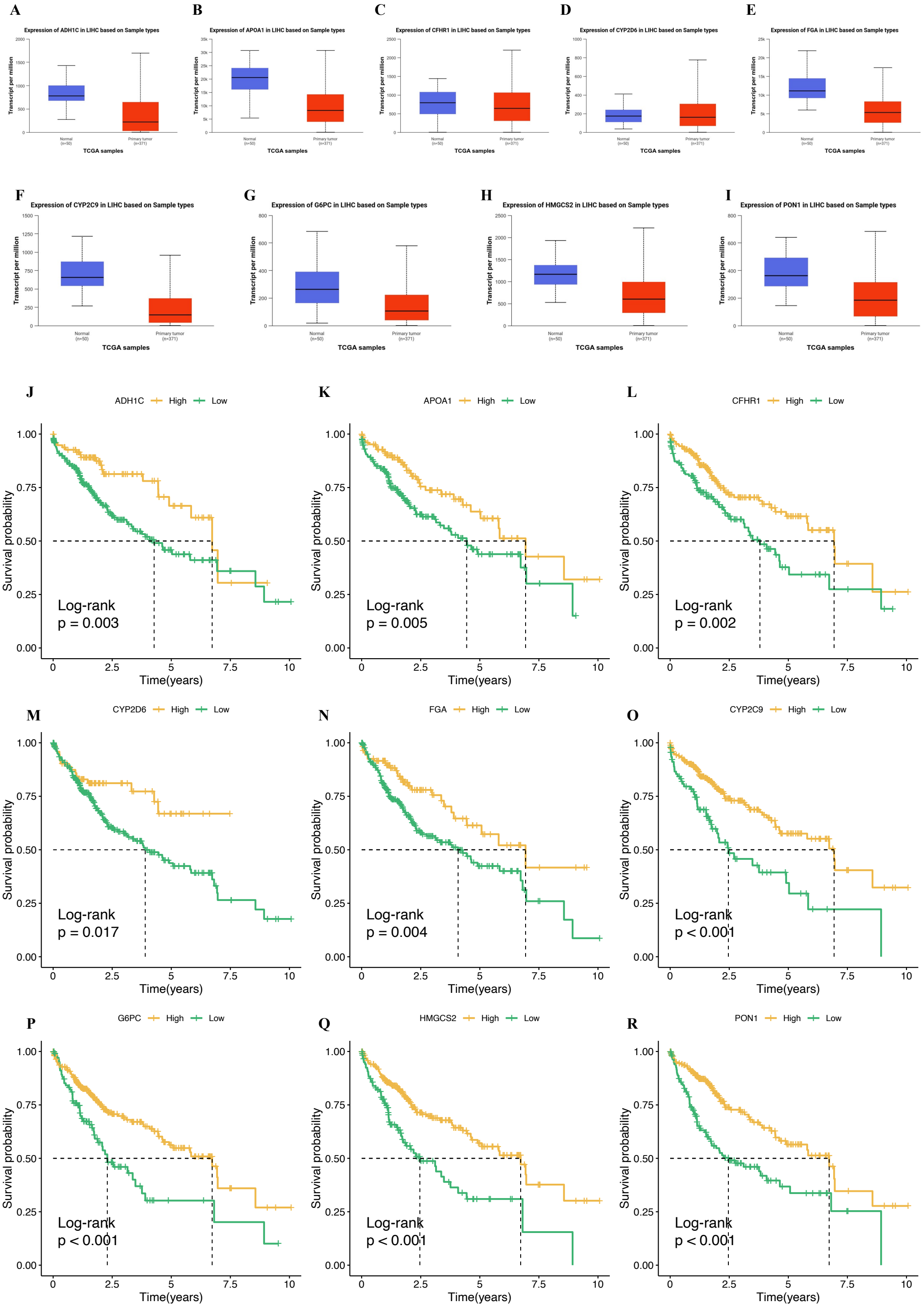

(A-I) The boxplot shows the differential expression of ADH1C (A), APOA1 (B), CFHR1 (C), CYP2D6 (D), FGA (E), CYP2C9 (F), G6PC (G), HMGCS2 (H), and PON1 (I) as analyzed by UALCAN.

(J-R) The Kaplan–Meier survival curve reveals survival differences in HCC patients based on the expression levels of the nine characteristic genes.
